# Supplementary material for: Atlas of breast cancer infiltrated B-lymphocytes revealed by paired single-cell RNA-sequencing and antigen receptor profiling
Source: Nat Commun. 2021 Apr 12;12:2186. doi: 10.1038/s41467-021-22300-2 (PMC8042001; doi:10.1038/s41467-021-22300-2)
Supplement: Supplementary file 1 — Supplementary Information [file 41467_2021_22300_MOESM1_ESM.pdf]

Supplementary Information for

Atlas of breast cancer infiltrated B-lymphocytes revealed by paired single-cell RNA-sequencing and antigen receptor profiling

Qingtao Hu[1, 5], Yu Hong[1, 2, 5], Pan Qi[3, 5], Guangqing Lu[1], Xueying Mai[1], Sheng Xu[3], Xiaoying He[3], Yu Guo[3], Linlin Gao[1], Zhiyi Jing[1], Jiawen Wang[1], Tao Cai[1], Yu Zhang[1,2,4*]

[1]National Institute of Biological Sciences, Beijing 102206, China;

[2]Peking University-Tsinghua University-National Institute of Biological Sciences Joint Graduate Program, School of Life Sciences, Peking University, Beijing 100871, China；

[3]Xinxiang central hospital, Xinxiang, Henan 453003, China;

[4]Tsinghua Institute of Multidisciplinary Biomedical Research, Tsinghua University, Beijing 100084, China;

[5]Theses authors contributed equally to this work.

*Correspondence should be addressed to Y. Z. (zhangyu@nibs.ac.cn).

This PDF file includes:

Supplementary Results and Methods for T cell clustering analysis for TNBC2-6 samples

Supplementary Figure 1-30

Supplementary Table 1-9

Supplementary References

Supplementary Results and Methods for T cell clustering analysis for TNBC2-6 samples

Supplementary Results

We analyzed 13,186 T cells from the five TNBC patients (TNBC2-6 and PBMC2-6) that had both RNA-seq data and a single assembled productive TCRα/TCRβ pair in tumor and peripheral blood samples. Unsupervised clustering of the combined T cells (TNBC2-6 and PBMC2-6) revealed 15 clusters with distinct transcriptional signatures (Supplementary Fig. 25-28). Cells from different tumor and blood samples contributed to each cluster, suggesting lack of sample batch effect and conserved differentiation process (Supplementary Fig**.** 27).

Nine groups of *CD4* T cell clusters were identified, including *CD4* Naive (C4, C6), *CD4* Treg (C2, C13), *CD4 CXCL13* (C1, C7, and C15), and *CD4 GZMK* (C10, C11). *CD4* Naive cells (C4 and C6) were mainly contributed by PBMC samples (p-value=0.06 and 0.03) and both showed high expressions of naive *CD4* markers such as *CCR7* and *TCF7*. Comparing with C6, C4 (*CD4* Naive *LEF1*^{HI}) showed higher expression of *LEF1*. *CD4* Treg cells (C2 and C13) expressed *FOXP3* and were dominantly presented in TNBC samples (p-value=0.02 and 0.09). Similar as in human bladder cancer[1], they could be further separated into *CD4* Treg *IL2RA*^{LO} (C2) and *CD4* Treg *IL2RA*^{HI} (C13). All *CD4 CXCL13* cells (C1, *CD4 CXCL13*; C7, *CD4 CXCL13 HSP*; C15, *CD4 CXCL13*^{LO}) showed strong expression of *CXCL13*, *IFNG*, *TOX*, *ICOS*, and *TIGIT*. C1 and C7 cells showed higher expression of T follicular helper (Tfh) signature[2] and Tfh-related genes (*MAF*, *SH2D1A*, *PDCD1*, *CD200*, *IFNG*, *IL2*, *CTLA4*) than C15. Comparing with C1, C7 cells showed higher expression of *FOS*/*JUN* and *HSP* family genes. C15 cells expressed lower level of *CXCL13* and higher levels of interferon pathway associated genes such as *IFI6*, *IFIT2*, *IFIT3*, *ISG15*, and *ISG20*. There were also two groups of cytotoxic *CD4 GZMK* T cells (C10, *CD4 GZMK* and C11, *CD4 GZMK EIF1*^{HI}). They both

expressed *GZMA* and *GZMK*, but not *GZMB* and *GNLY*. C11 was dominantly in TNBC samples (p-value= 0.01). Comparing with C10, only C11 cells showed expression of *PRF1* and *NKG7*.

Five *CD8* T cell clusters were identified, including *CD8* Naive (C14), *CD8* Exhausted (C3), *CD8* Effector (C5), *CD8 FOS*(C8), and CD8 *XCL* (C9). *CD8* Naive cells (C14) were mainly contributed by PBMC samples (p-value=0.07) and showed high expressions of naive *CD8* markers such as *LEF1*, *CCR7*, and *SELL*. *CD8* Effector cells (C5) were also mainly contributed by PBMC samples (p-value=0.03) and showed high expressions of *CX3CR1*, *FGFBP2*, and *GNLY*. *CD8* Exhausted cells (C3) represented exhausted tumor reactive *CD8* T cells[3] and were positive for *HACXR2*, *LAG3*, *CXCL13*, and *PDCD1*. They showed the strongest expression of Trm signature[4]. *CD8 FOS* cells (C8) were mainly contributed by TNBC samples (p-value=0.04). In addition, we also identified *CD8 XCL* (C9) cells with high expression of *XCL1/2*, *TRGC2*, *NUCB2*, and *NCR3*[1].

C12 *CD4*/*CD8* positive cells were mixed with *CD4* and *CD8* positive cells at current clustering resolution. They had high expression of proliferation markers such as *PCNA* and *KI67*. They were mainly contributed by TNBC samples (p-value= 0.01).

Analysis of the single productive TCR rearrangements from 10,972 infiltrated T cells in TNBCs and 5,737 T cells from their corresponding PBMCs did not reveal significantly different VH and JH gene usage for majority of them (Supplementary Fig**.** 29a-b). As shown in Supplementary Fig. 29c, TNBC-infiltrated T cells contained higher percentages of T cell clones than PBMC samples (70% vs 34%), which were also larger than the clones in PBMCs (Supplementary Fig. 29c-d).

To demonstrate the intra- and inter-cluster distributions of T cell clones, the overlap of TCR clonotypes between each two clusters was shown in a heat map after normalized by their cell numbers (Supplementary Fig. 30). In tumor tissues, we found that *CD8* Exhausted (C3) had the most intra-cluster

clonotype overlap, suggesting the highest clonality for this group (Supplementary Fig. 30a). CD8 Exhausted (C3) and *CD4*/*CD8* (C12) clusters had the most inter-cluster clonotype overlap. In PBMC, significantly less clonotype overlap was observed than in TNBC samples (Supplementary Fig. 30b).

Supplementary Methods

T cell clustering analysis for TNBC2-6 samples

After processing following the 'Quality control and filtering of single-cell RNA-seq data' section, T cells with a single TCRα and TCRβ pair in TCR libraries and BCR negativity in BCR libraries were selected for T cell subgrouping analysis using Seurat[5] (13,186 cells for TNBC2-TNBC6 samples). For those T cells in each library, library-size normalization to each cell was performed by NormalizeData. The variability of the numbers of UMIs was regressed out by ScaleData. The variable genes were calculated by FindVariableGenes. RunMultiCCA was used to combine the T cells from all libraries, and 15 CC dimensions were used for AlignSubspace. FindClusters was used to cluster cells using the 15 aligned CCs at a resolution of 1.5 for grouping (Supplementary Fig. 25-26). The CC number was determined by inspecting the results of DimHeatmap. The clustering results were visualized with t-SNE dimensionality reduction using RunTSNE (15 aligned CCs) and TSNEPlot. The marker genes of each cluster were detected by the FindAllMarkers function with the default parameters, except min.pct was set to 0.25. The genes were ranked by their fold-change (from largest to smallest), and the top 10 marker genes of each cluster were shown in the heatmap in Supplementary Fig. 26.

Paired TCR and single-cell RNA-seq data analyses

To demonstrate the intra- and inter-cluster distributions of T cell clones, we

counted the overlap of clonotypes between two T cell clusters. T cell clonotype was defined as cells with the same CDR3 nucleotide sequences for TCRα and TCRβ pair. For any two cells with the same clonotype, the overlap number between the two T cell clusters containing those two cells was added with 1. Then there would be a sum number of overlaps for each two T cell clusters. To compare the levels of the overlap among T cell clusters with different cell numbers, the counts of overlap were normalized by the formula (2) $N_{normalized} = \frac{N}{N_A} \times \frac{N}{N_B} \times 10{,}000$ in the heatmap (Supplementary Fig. 30a-b). $N_{normalized}$ is the normalized value shown in Supplementary Fig. 30a-b. N is the sum number of overlaps for two T cell clusters, and N_A and N_B are the cell numbers of the two T cell clusters. Then, heatmaps were generated for PBMCs (when two cells came both from PBMC samples) and TNBCs (when two cells came both from TNBC samples).

Supplementary Figures

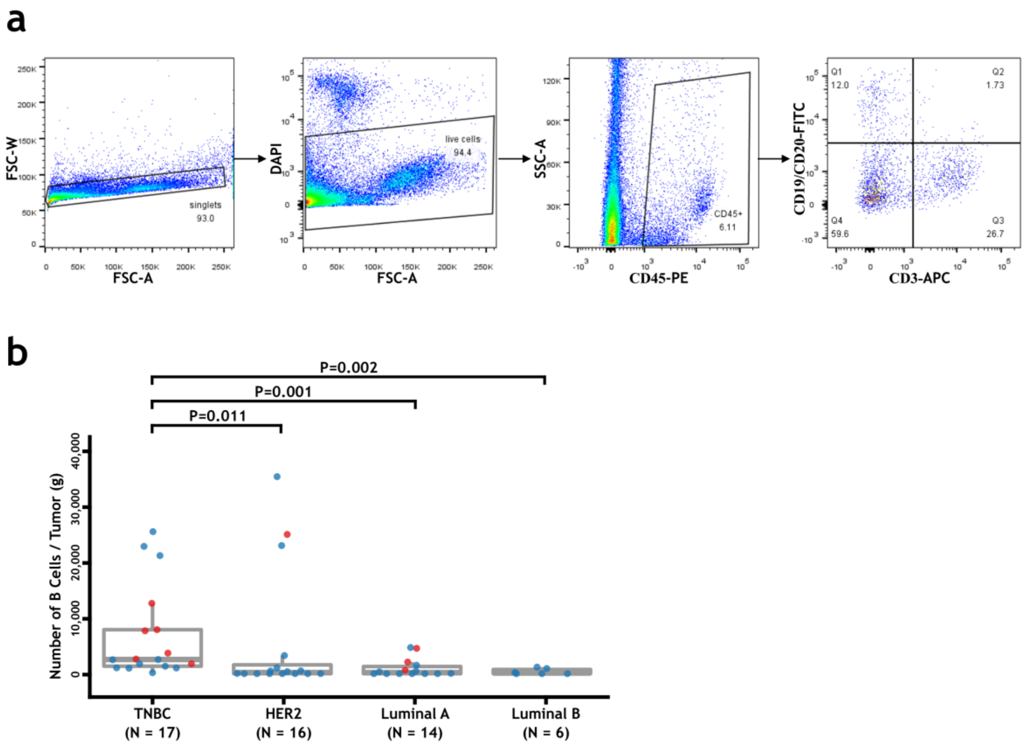

Supplementary Fig. 1. TNBC tumors have more infiltrated B cells than other breast cancer types

(a) Gating strategy for sorting of $CD45^+$ cells, B cells ($CD45^+CD19/CD20^+CD3^-$), and T cells ($CD45^+CD3^+CD19/CD20^-$). DAPI was used to enrich living cells. Sorting for B cells ($CD45^+CD19/CD20^+CD3^-$) was used in Supplementary Fig. 1b.

(b) The numbers of B cells in breast tumors quantified by FACS were normalized to tumor weight (per gram). The p-values were calculated by the Wilcoxon rank sum test (unpaired, two-sided). Red dots represent samples for single-cell sequencing. The sample numbers for FACS analysis were n=17 (TNBC), n=16 (HER2), n=14 (Luminal A), and n=6 (Luminal B). Box plots show the median (center bar), the third and first quartiles (upper and lower edges of the box, respectively). Limits of the upper and lower whiskers in the box plots were the largest and smallest values that are $\leqslant$1.5 times the interquartile range, respectively.

Source data are provided as a source data file.

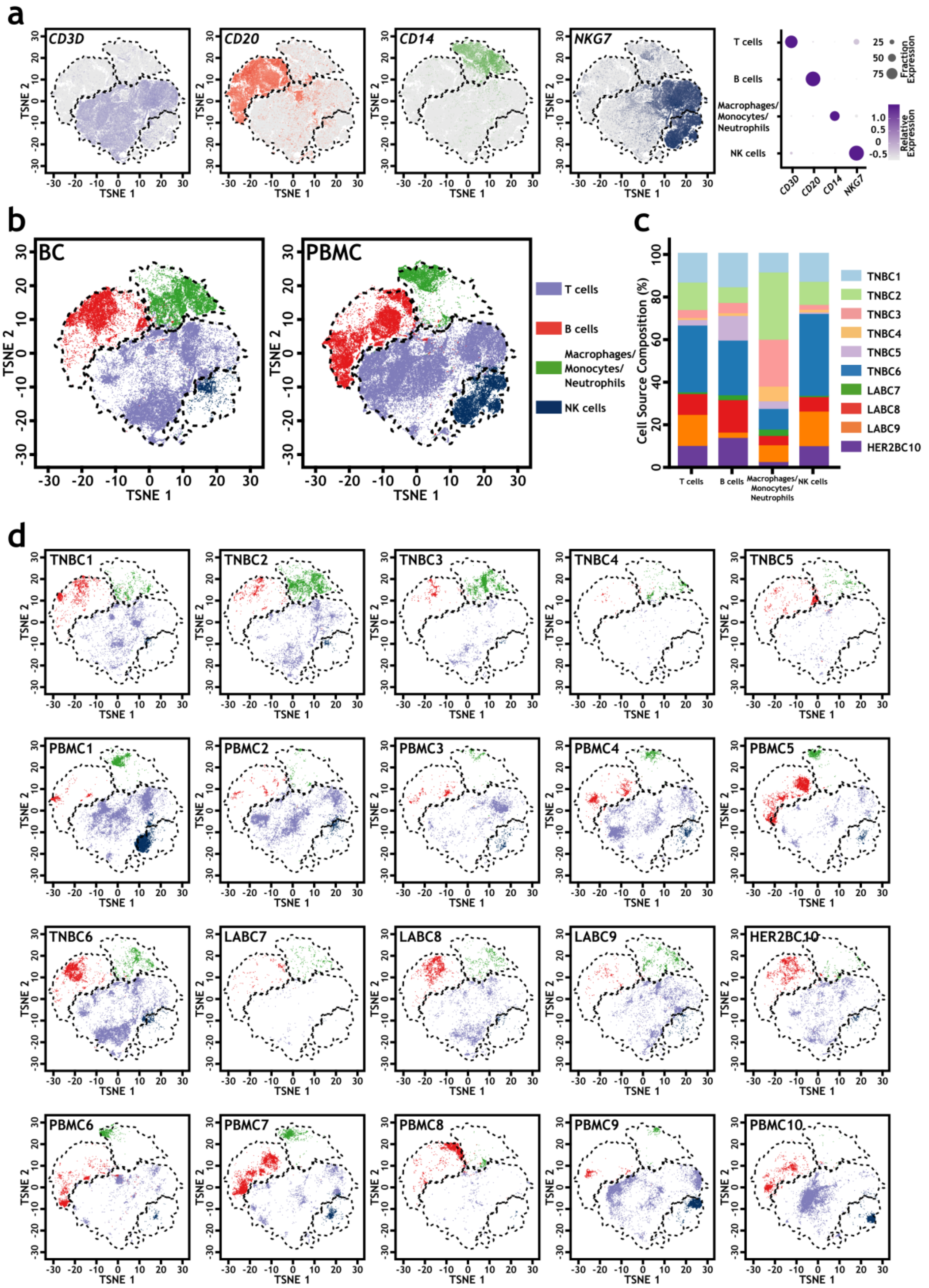

Supplementary Fig. 2. Unsupervised clustering of all sequenced samples at low resolution reveals four major cellular clusters, including T cells, B cells, NK cells, and macrophages/monocytes/neutrophils

(a) The t-SNE projections of marker genes for T cells (*CD3D*), B cells (*CD20*), NK cells (*NKG7*), and macrophages/monocytes/neutrophils (*CD14*).

(b) The t-SNE projections of TNBC cells (left panel) and PBMCs (right panel) separately.

Both TNBC and PBMC samples contribute similarly to each cluster.

(c) Cells from different tumor samples contributed similarly to each cell cluster (also see Supplementary Table 4).

(d) The t-SNE projections of *CD45*$^+$ cells from each sample.

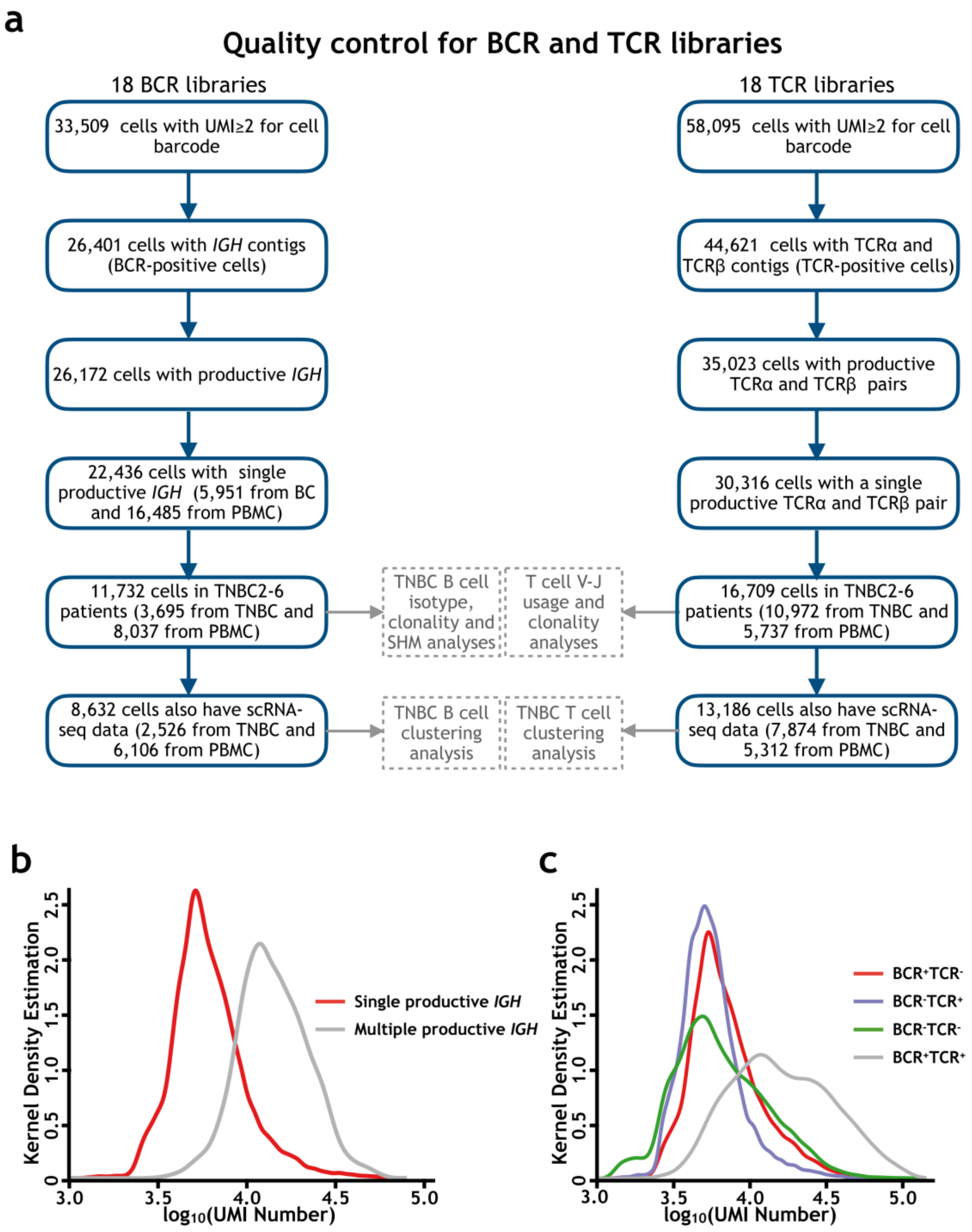

Supplementary Fig. 3. Quality control steps for BCR/TCR library analyses

(a) Quality control steps for BCR libraries and TCR libraries.

(b) There are significantly more unique molecular identifiers (UMIs) in cells with multiple productive *IGH*s than in cells with a single productive *IGH*. X is the $\log_{10}$ UMI count observed in cells. Y is the kernel density estimation.

(c) There are significantly more UMIs in BCR and TCR double-positive cells than in BCR-positive cells, TCR-positive cells and BCR/TCR double-negative cells. X is the $\log_{10}$ UMI count observed in cells. Y is the kernel density estimation.

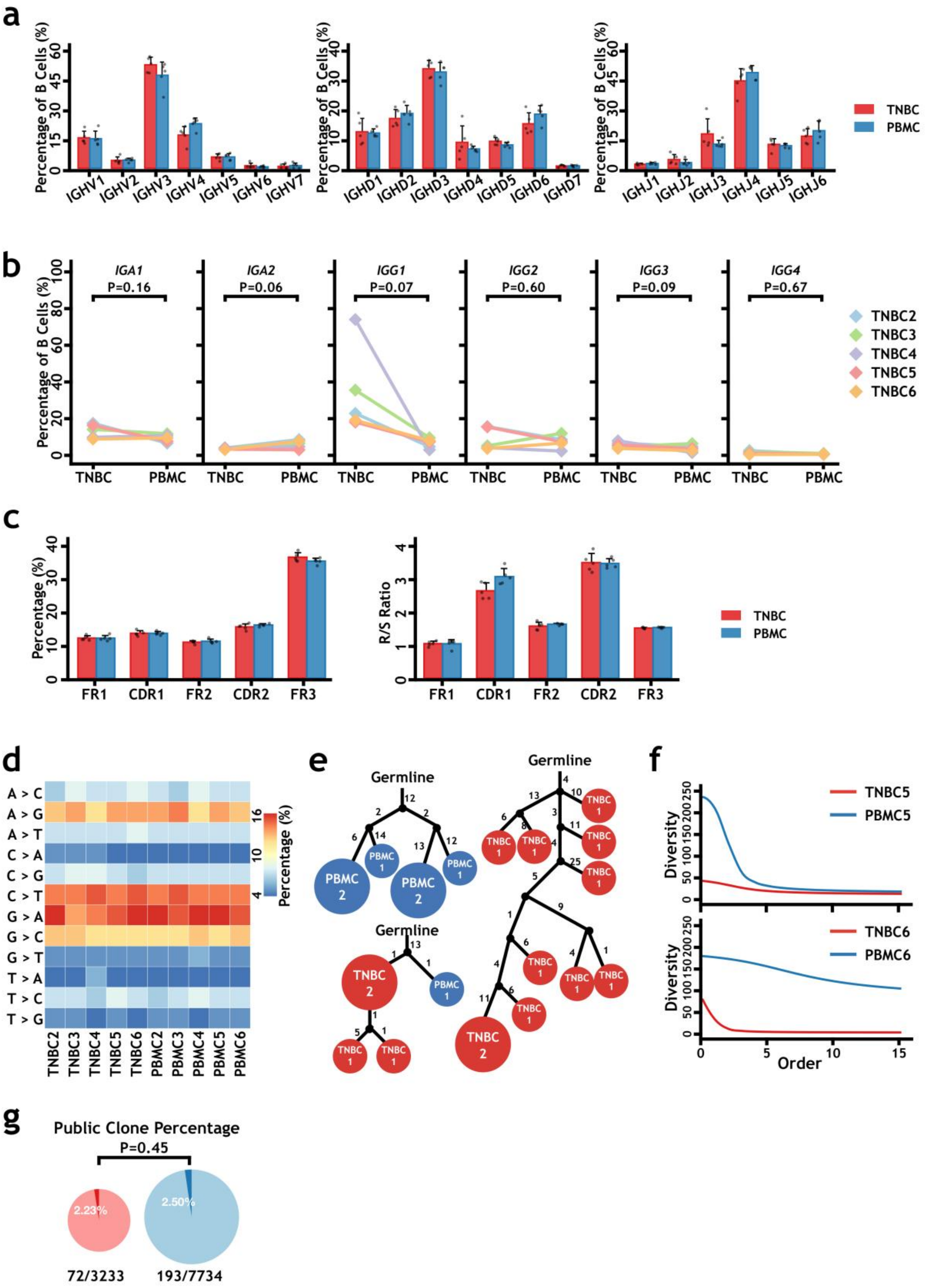

Supplementary Fig. 4. Single-cell *IGH* analysis of B cells in tumor and peripheral blood samples from TNBC patients

(a) VH, DH, and JH usage in TNBC (red, n=5, TNBC2-6) and PBMC (blue, n=5, PBMC2-6). Error bars represent the mean±standard deviation (SD) of the five samples. No significant difference was observed between TNBC and PBMC samples (a two-tailed paired Student's t-test was used, and the threshold for significance was set at p<0.05. P

values were 0.67, 0.75, 0.27, 0.10, 0.98, 0.25, 0.65 for the left panel, 0.88, 0.07, 0.58, 0.43, 0.11, 0.09, 0.88 for the middle panel, and 0.13, 0.24, 0.24, 0.14, 0.67, 0,09 for the right panel, from left to right).

(b) Distributions of *IGH* isotypes in TNBC and PBMC samples. The p-values were calculated by two-tailed paired Student's t-test.

(c) Comparison of SHMs in different *IGH* V(D)J regions. Left panel, mutation distributions in different V(D)J regions. Right panel, replacement mutation/silent mutation (R/S) ratio comparison in different V(D)J regions. No significant difference was observed between TNBC (n=5, TNBC2-6) and PBMC (n=5, PBMC2-6) (a two-tailed paired Student's t-test was used, and the threshold for significance was set at $p<0.05$. P values were 0.96, 1, 0.48, 0.23, 0.08 for the left panel and 0.96, 0.07, 0.28, 0.86, 0.07 for the right panel, from left to right). Error bars represent the mean±standard deviation (SD) of the five samples.

(d) *IGH* SHM signatures of each sample. Threshold for significance was set at $p<0.05$ and only G>T reached the threshold with $p=0.02$, two-tailed paired Student's t-test.

(e) Examples of B cell clonal trees with only PBMC cells, only TNBC cells, and both PBMC and TNBC cells. The trees are rooted in the closest *IGH* V(D)J germline allele in the IMGT database. Numbers indicate somatic mutations. Red circles represent TNBC-infiltrated B cells, and blue circles represent PBMC B cells. The sizes of the circles are proportional to cell numbers (also indicated in the circles). Black dots indicate inferred nodes.

(f) The *IGH* diversities of TNBC5 and TNBC6 samples are lower than those of the corresponding PBMC samples. The diversity of clones with at least 2 cells is plotted at different orders (Hill numbers). At an order of 0, the diversity is the number of different clones. At orders >1, the diversity is influenced more by the most abundant clones[33].

(g) The percentage in the pie represents the number of public clones between those in the current study and those in the dataset from Briney et al[6]. divided by the number of clones in TNBC and PBMC, and the numbers are presented under the pie charts. The p-value was calculated by two-sided Fisher's exact test using the number of public clones and non-public clones in TNBC and PBMC.

Source data are provided as a source data file.

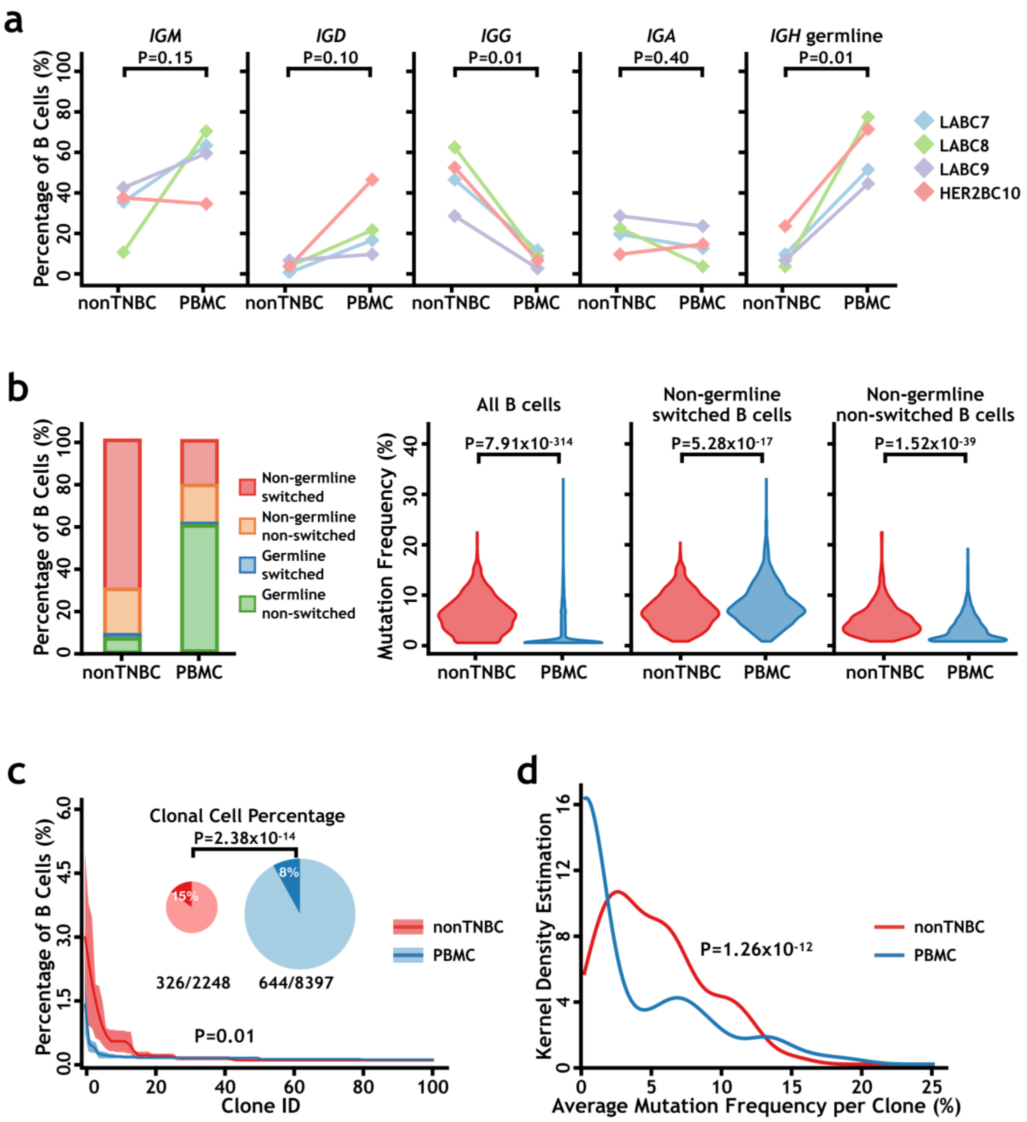

Supplementary Fig. 5. Single-cell *IGH* analysis of B cells in tumor and peripheral blood samples from LABC and HER2BC patients

(a) Distributions of *IGH* isotypes and germline *IGH* in B cells from non-TNBC (LABC7-9 and HER2BC10) and corresponding PBMC samples. The p-values were calculated by two-tailed paired Student's t-test.

(b) Comparisons of CSR and SHM between B cells from non-TNBC (LABC7-9 and HER2BC10) samples and those from corresponding PBMC samples. The left bar plot presents the percentages of cells that have germline or non-germline and class-switched or non-switched *IGH* alleles. The violin plots from left to right show the comparisons of SHM rates for all B cells, for non-germline and class-switched B cells, and for

non-germline and non-switched B cells. The p-values were calculated by two-tailed Student's t-test.

(c) The non-TNBC (LABC7-9 and HER2BC10) tumor samples contained significantly more and larger B cell clones. The percentages of clonal B cells in all B cells are presented by the upper pie charts, and the p-value was calculated by two-sided Fisher's exact test. The size distribution of B cell clones showed that non-TNBC breast cancer (LABC and HER2BC) samples had larger B cell clones than PBMC samples, and the p-value was calculated by two-tailed Student's t-test.

(d) The non-TNBC (LABC7-9 and HER2BC10) tumor clones had a higher *IGH* mutation frequency than the PBMC clones. The average *IGH* mutation frequencies of each clone are plotted versus the percent of clones with that mutation level. The p-value was calculated by two-sided Kolmogorov-Smirnov test.

Source data are provided as a source data file.

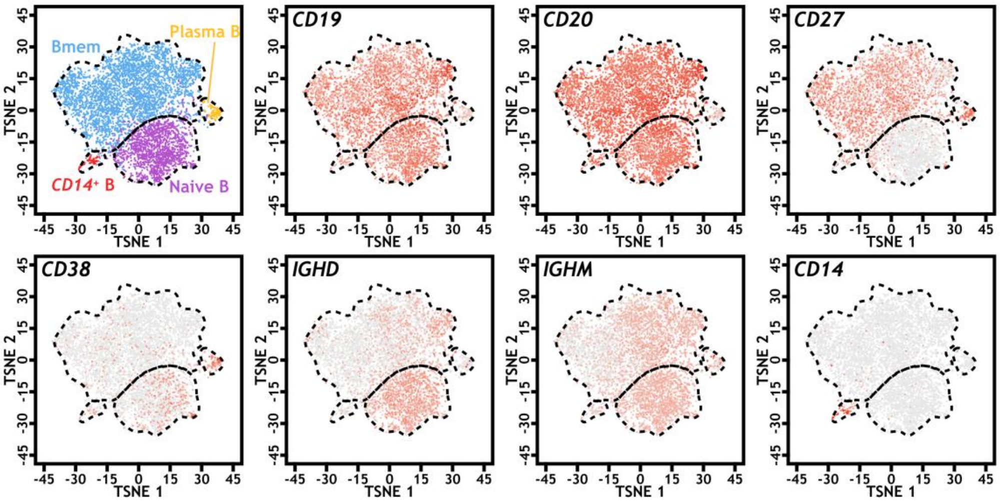

Supplementary Fig. 6. B cell groups defined by marker gene expression patterns

The 4 major B cell clusters (upper left panel) and the t-SNE projections of marker genes for the naive B cell (*IGM*$^{+}$*IGD*$^{+}$), memory B cell (*CD27*$^{+}$*CD38*$^{-}$), plasma B cell (*CD27*$^{+}$*CD38*$^{+}$), and *CD14*$^{+}$ B cell groups. Each dot represents a cell. The color scale represents the normalized expression levels of those genes. The red color represents higher gene expression.

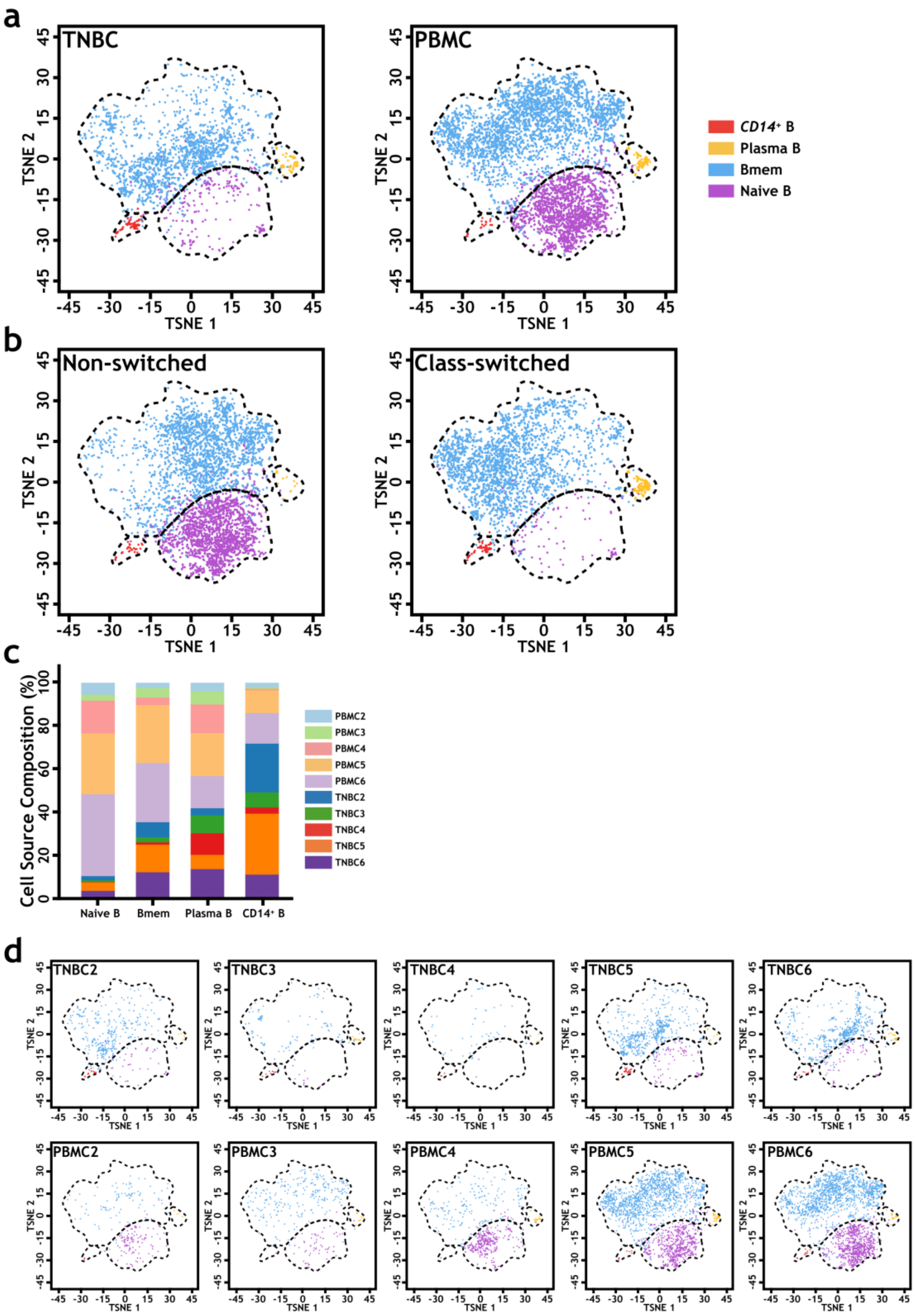

Supplementary Fig. 7. Unsupervised clustering of B cells from TNBC patients at low resolution revealed 4 different cell clusters

(a) The t-SNE projections of TNBC B cells (left panel) and PBMC B cells (right panel). Both TNBC and PBMC samples contributed similarly to each cluster.

(b) The t-SNE projections of *IGH* non-switched B cells (left panel) and *IGH* class-switched B cells (right panel). *IGH* class-switched and non-switched B cells could be well separated in the t-SNE plots.

(c) Percentages of cells from each sample for each B cell cluster. Cells from different samples contributed similarly to each B cell cluster (also see Supplementary Table 5).

(d) The t-SNE projections of B cells from each sample.

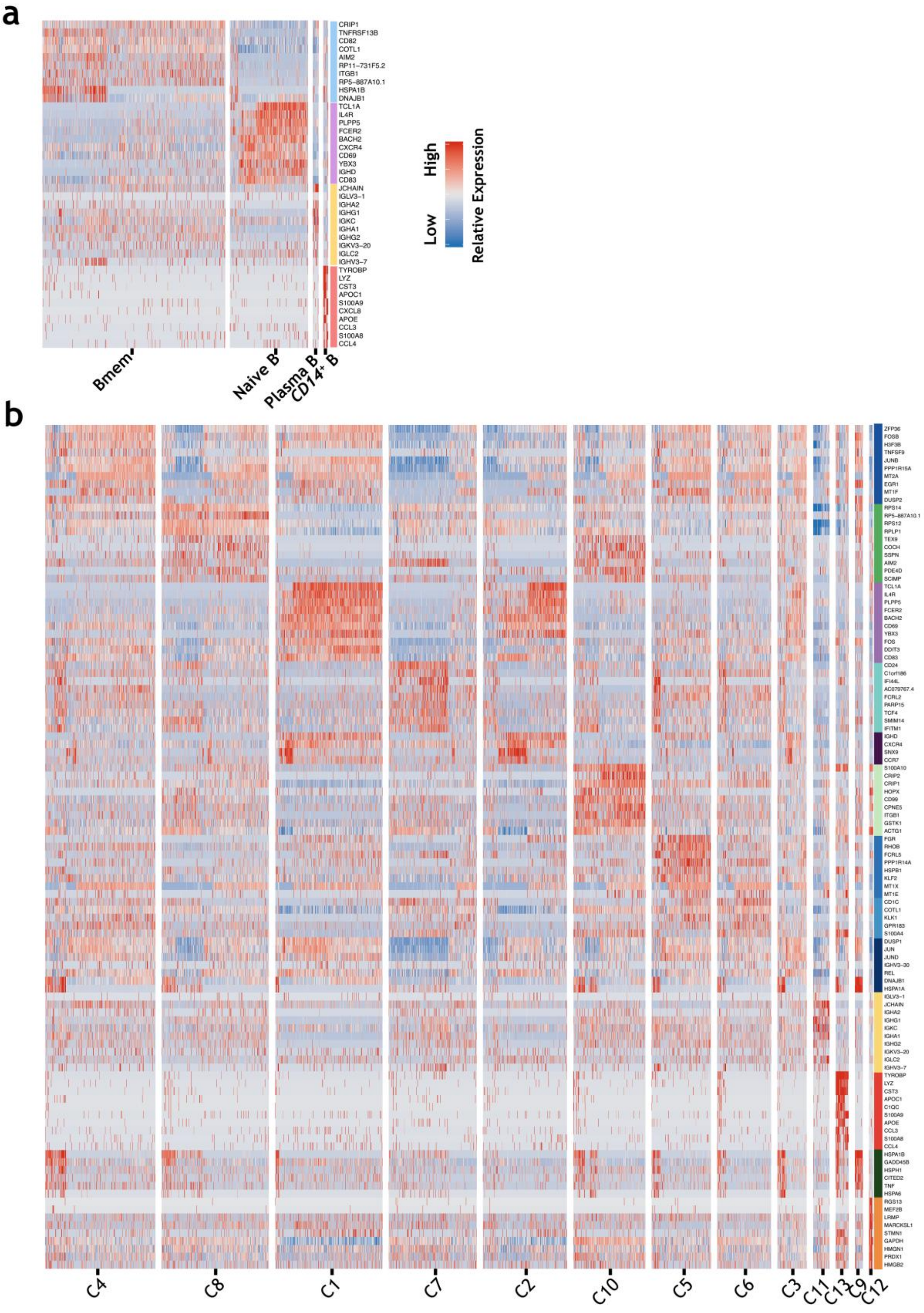

Supplementary Fig. 8. Heat maps of marker genes in each B cell group

(a) Heat map of the top 10 marker genes for the 4 B cell clusters from low-resolution grouping. Gene names are labeled alongside. Red represents a high expression level, and blue represents a low expression level (also see Supplementary Data 2).

(b) Heat map of the top 10 marker genes for the 13 B cell clusters from high-resolution

grouping. Gene names are labelled alongside. Red represents a high expression level, and blue represents a low expression level (also see Supplementary Data 3).

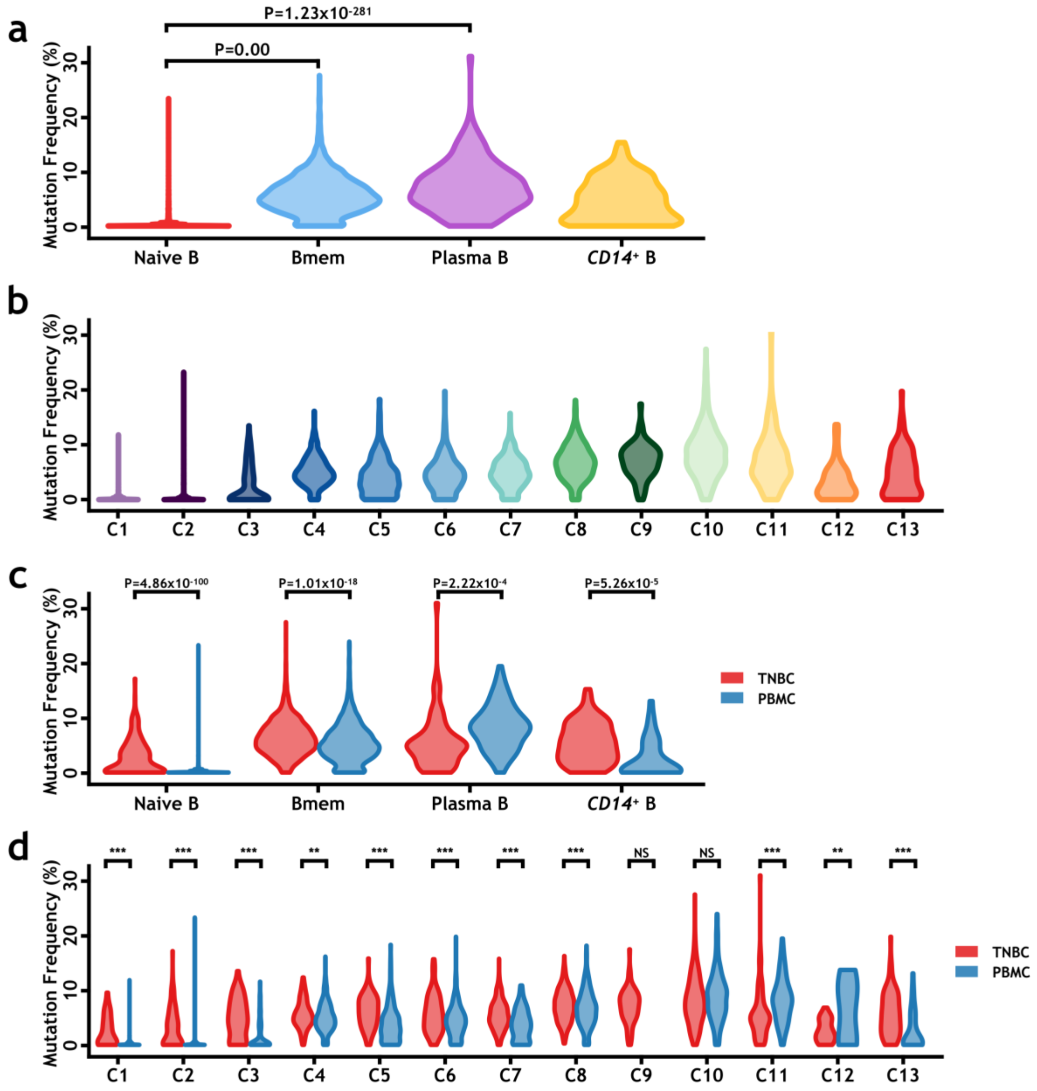

Supplementary Fig. 9. Comparisons of SHM frequencies among different B cell clusters

(a) SHM frequencies of cells in the four B cell clusters. The p-values were calculated by two-tailed Student's t-test.

(b) SHM frequencies of cells in the 13 B cell clusters.

(c) Comparisons of the SHM frequencies between TNBC B cells and PBMC B cells in the 4 B cell clusters. The p-values were calculated by two-tailed Student's t-test.

(d) Comparisons of the SHM frequencies between TNBC B cells and PBMC B cells in the 13 cell clusters. There was only one cell in C9 from PBMCs. The p-values were calculated by two-tailed Student's t-test. ** means p-value < 0.01; *** means p-value < 0.001; NS

means p-value > 0.05. The exact p values were 1.16×10^{-48} (C1), 1.94×10^{-66} (C2), 1.46×10^{-18}(C3), 2.47×10^{-3}(C4), 1.57×10^{-10}(C5), 4.00×10^{-5}(C6), 9.79×10^{-20}(C7), 7.60×10^{-4}(C8), 0.31(C9), 0.07(C10), 6.48×10^{-5}(C11), 4.47×10^{-3}(C12), and 1.02×10^{-5}(C13).

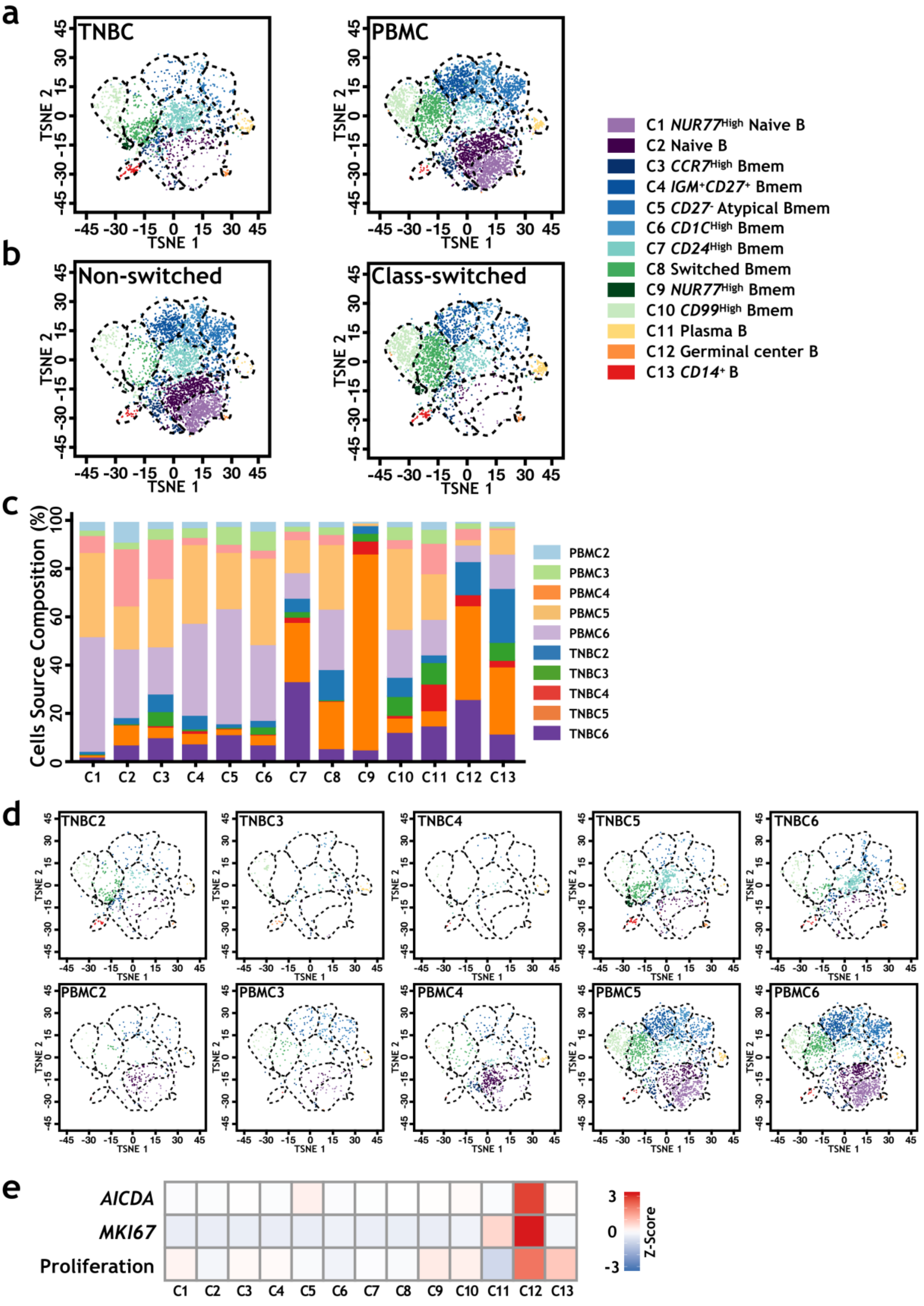

Supplementary Fig. 10. Unsupervised clustering of B cells from TNBC patients at higher resolution revealed 13 different cell clusters

(a) The t-SNE projections of TNBC B cells (left panel) and PBMC B cells (right panel). Both TNBC and PBMC samples contributed similarly to each cluster.

(b) The t-SNE projections of *IGH* non-switched B cells (left panel) and *IGH* class-switched

B cells (right panel). *IGH* class-switched and non-switched B cells could be well separated in the t-SNE plots.

(c) Percentages of cells from each sample for each B cell cluster. Cells from different samples contributed similarly to each B cell cluster (also see Supplementary Table 6). C12 germinal center B cells were observed mainly in tumor samples and in 4 of the 5 patients (also see Supplementary Table 6).

(d) The t-SNE projections of B cells from each sample.

(e) Rows represent averaged expression values of activation-induced cytidine deaminase (*AICDA*) (upper panel) and *MKI67* (middle panel) and averaged proliferation scores (lower panel). Each column represents one B cell cluster. The color scheme is based on the z-score distribution (see 'Heatmaps of relative gene expression and signature scores for each cluster' in the Methods section for details).

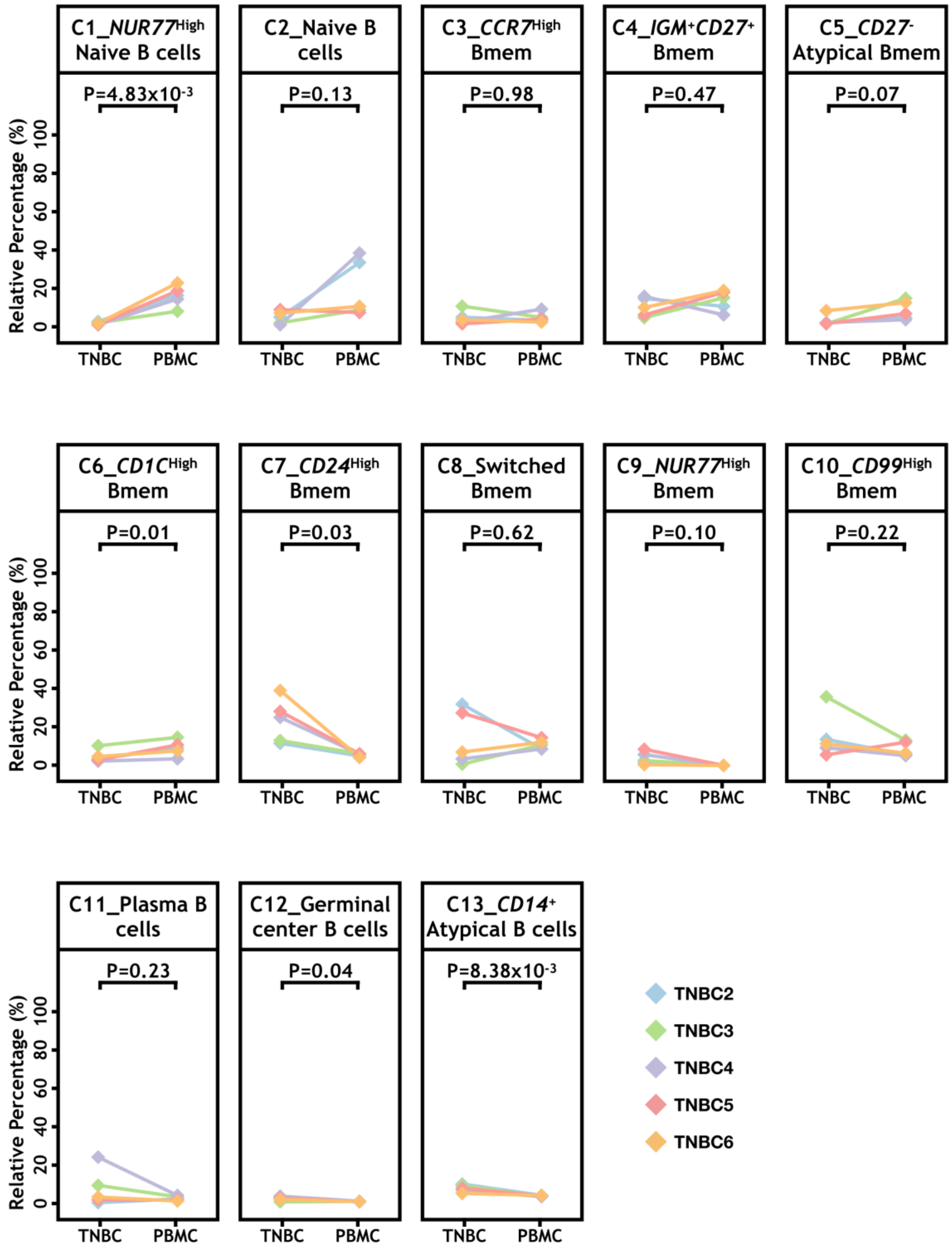

Supplementary Fig. 11. The relative percentages of different B cell clusters in TNBC and PBMC samples

The distributions of 13 clusters in each sample. The p-values were calculated by two-tailed paired Student’s t-test.

Source data are provided as a source data file.

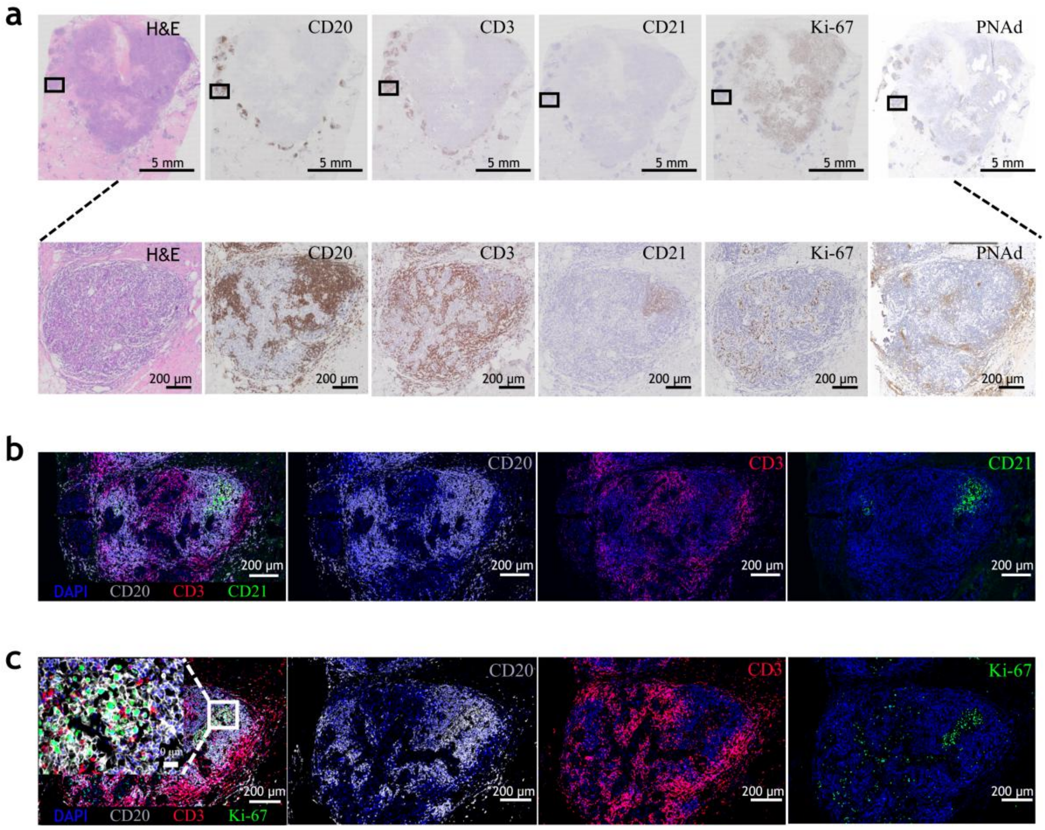

Supplementary Fig. 12. Tertiary lymphoid structures and putative germinal center B cells were identified in TNBC tumors.

(a) Representative images of H&E stainings and immunohistochemical (IHC) stainings of TNBC tumors for CD20, CD3, CD21, Ki-67, and PNAd expression. Lower panel showed H&E staining and IHC stainings for indicated markers of boxed area in upper panel. The H&E stainings and IHC of CD20 and CD3 were conducted for TNBC1-6, and IHC of CD21, Ki-67, and PNAd were conducted for TNBC1, TNBC5, and TNBC6. Similar results were observed and representative images from TNBC5 were showed.

(b) Multiplex immunofluorescence stainings of CD20, CD3, and CD21 of boxed area in **(a).** These multiplex immunofluorescence stainings were conducted for TNBC1 and TNBC5. Similar results were observed, and representative images from TNBC5 were showed.

(c) Multiplex immunofluorescence stainings of CD20, CD3, and Ki-67 of boxed area in **(a)**. These multiplex immunofluorescence stainings were conducted for TNBC1 and TNBC5. Similar results were observed, and representative images from TNBC5 were showed.

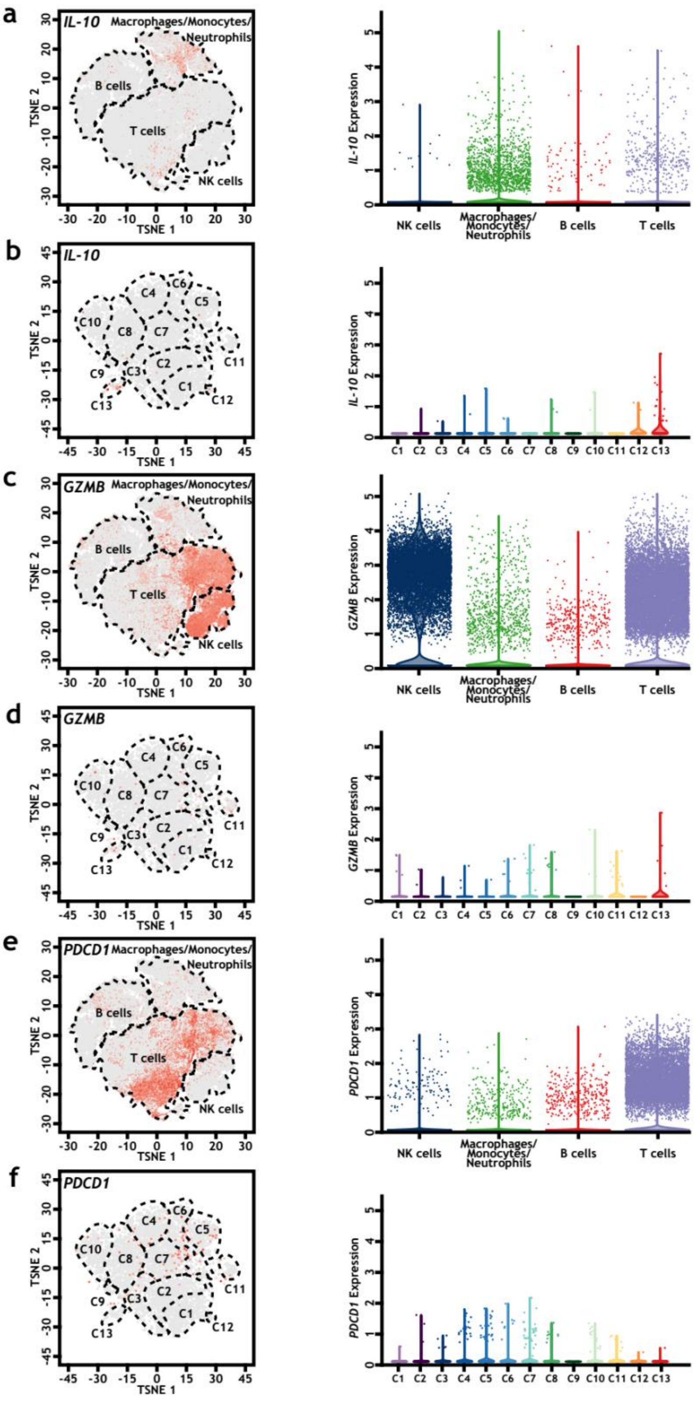

Supplementary Fig. 13. Expression patterns of *IL-10*, *GZMB, and PDCD1* in *CD45*$^+$ lymphocytes

(a) The t-SNE projection (left panel) and violin plot (right panel) of *IL-10* expression in *CD45*$^+$ cells from all samples. In the t-SNE plot, each dot represents a cell. The red color represents cells expressing *IL-10*. In the violin plot, the colors represent cell types, the same as in Fig 1b.

(b) The t-SNE projection (left panel) and violin plot (right panel) of *IL-10* expression in B cells from TNBC patient samples. In the t-SNE plot, each dot represents a cell. The red color represents cells expressing *IL-10*. In the violin plot, the colors represent cell types, the same as in Fig 4a.

(c) The t-SNE projection (left panel) and violin plot (right panel) of *GZMB* expression in *CD45*$^+$ cells from all patient samples. In the t-SNE plot, each dot represents a cell. The red color represents cells expressing *GZMB*. In the violin plot, the colors represent cell types, the same as in Fig 1b.

(d) The t-SNE projection (left panel) and violin plot (right panel) of *GZMB* expression in B cells from TNBC patient samples. In the t-SNE plot, each dot represents a cell. The red color represents cells expressing *GZMB*. In the violin plot, the colors represent cell types, the same as in Fig 4a.

(e) The t-SNE projection (left panel) and violin plot (right panel) of *PDCD1* expression in *CD45*$^+$ cells from all patient samples. In the t-SNE plot, each dot represents a cell. The red color represents cells expressing *PDCD1*. In the violin plot, the colors represent cell types, the same as in Fig 1b.

(f) The t-SNE projection (left panel) and violin plot (right panel) of *PDCD1* expression in B cells from TNBC patient samples. In the t-SNE plot, each dot represents a cell. The red color represents cells expressing *PDCD1*. In the violin plot, the colors represent cell types, the same as in Fig 4a.

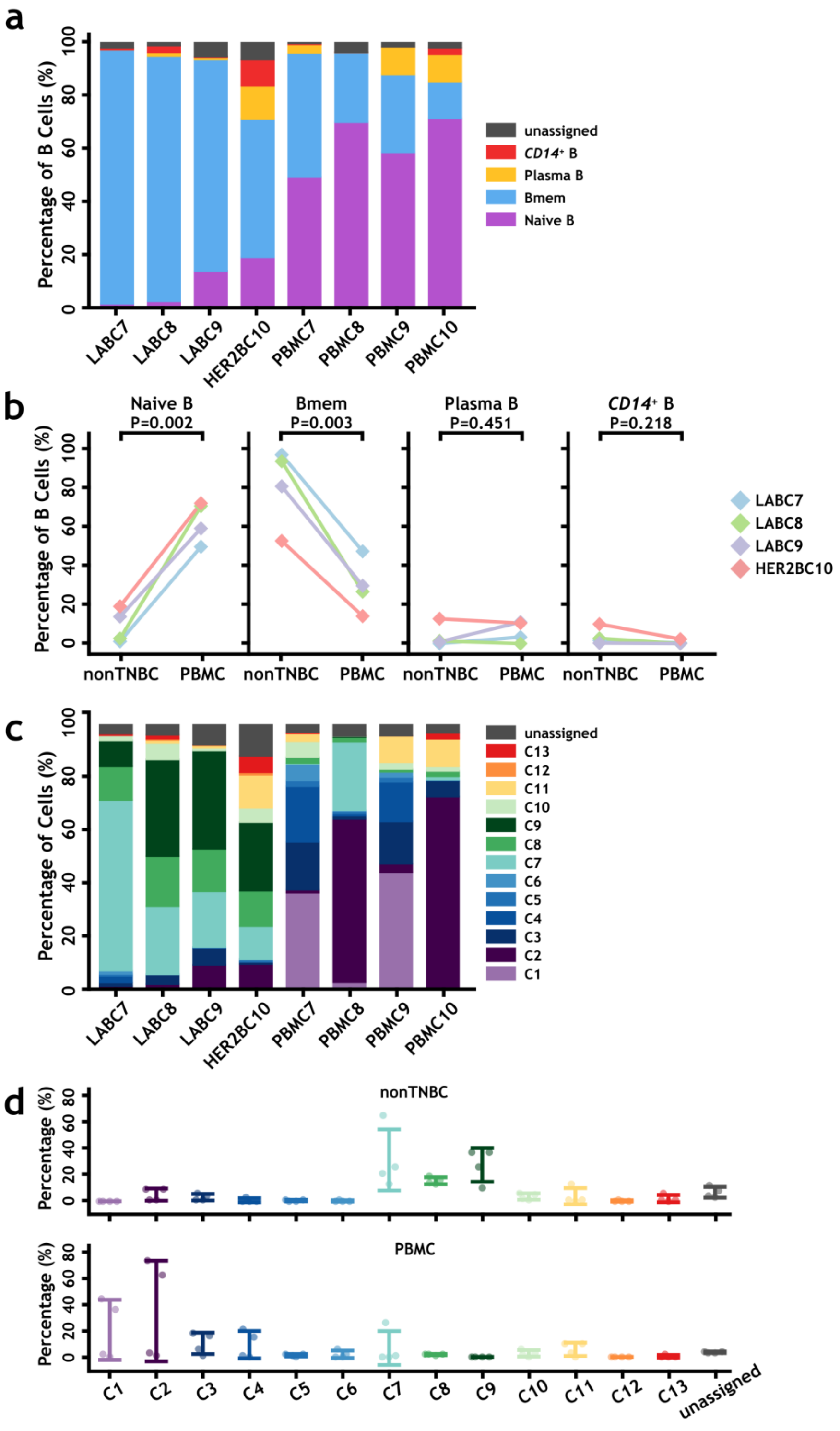

Supplementary Fig. 14. B cell subgroup analysis of non-TNBC patient (LABC7-9 and HER2BC10) samples

(a) Proportions of cells from LABC and HER2BC patients that were mapped to TNBC B cell clusters (4 clusters at low resolution).

(b) The distributions of the 4 clusters in each patient sample. The p-values were calculated by two-tailed paired Student's t-test.

(c) Proportions of cells from LABC and HER2BC patients that mapped to TNBC patient B cell clusters (13 clusters at high resolution).

(d) The distributions of the 13 clusters in each patient sample (upper panel: LABC and HER2BC tumors; lower panel: PBMC). X represents B cell clusters, and Y represents the cell percentages in each sample. Each dot represents one sample, and error bars represent the mean±standard deviation (SD) of the four samples.

Source data are provided as a source data file.

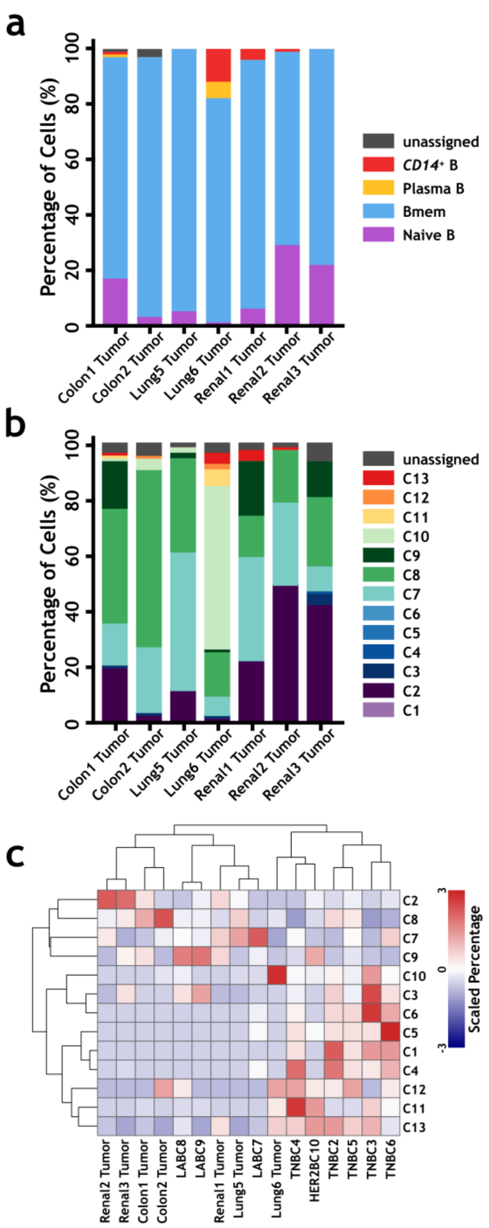

Supplementary Fig. 15. B cell subgroup analysis of tumor-infiltrated B cells in breast cancer, colon cancer, lung cancer, and renal cancer

(a) Proportions of infiltrated B cells from colon tumors, lung tumors, and renal tumors that were mapped to TNBC B cell clusters (4 clusters at low resolution).

(b) Proportions of infiltrated B cells from colon tumor, lung tumor, and renal tumor that were mapped to TNBC B cell clusters (13 clusters at high resolution).

(c) Hierarchical clustering of the relative percentages of 13 TIL-B cell subpopulations. TIL-B cell subpopulations are in rows, and subjects are in columns. Variable coloring is

based on the scaled percentages across subjects (removing the mean and dividing by the standard deviation). Clustering was conducted by the pheatmap R package using correlation similarity and complete linkage. Unassigned B cells were excluded before the percentage calculation.

Source data are provided as a source data file.

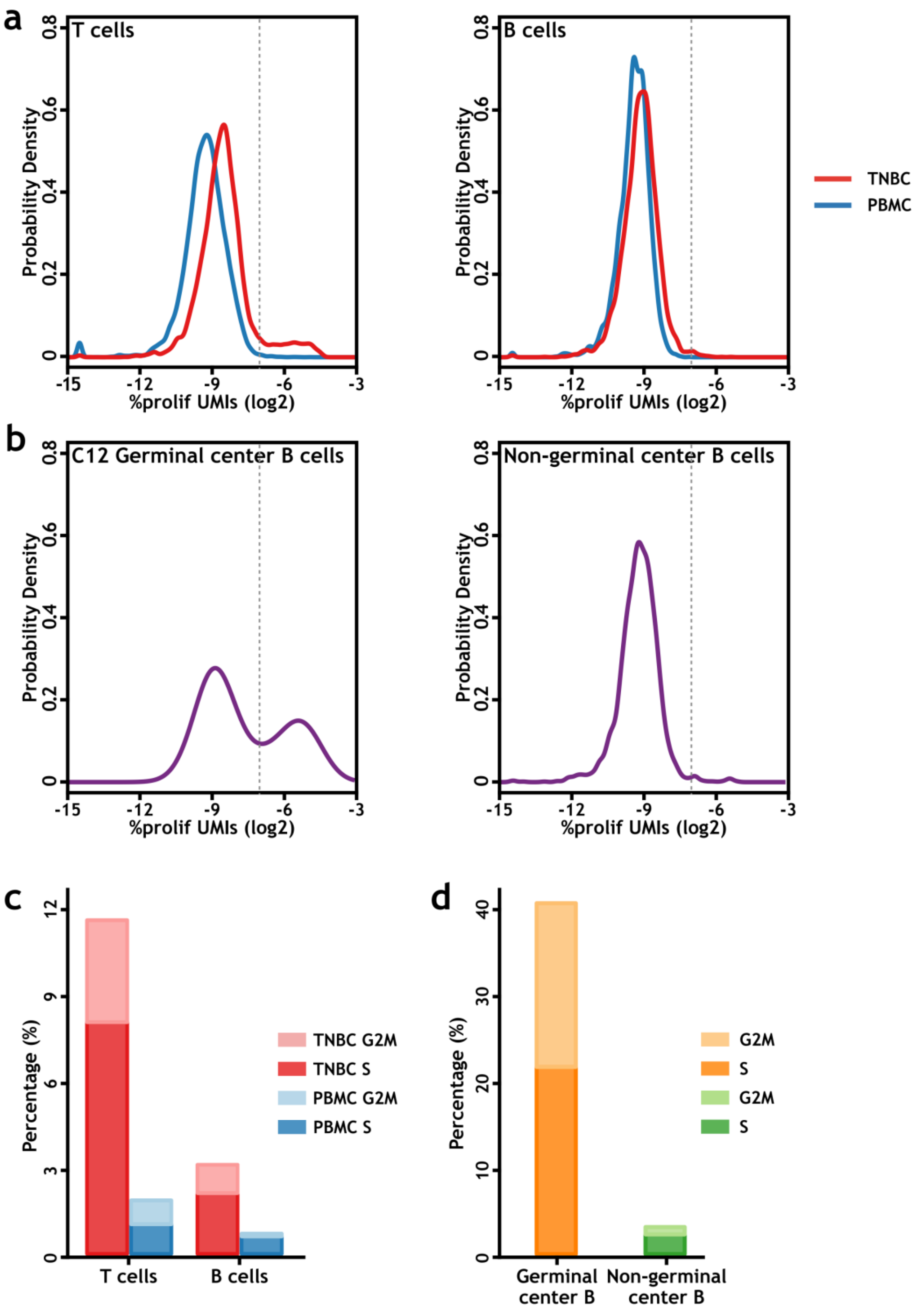

Supplementary Fig. 16. Cell cycle analysis of tumor-infiltrated B and T cells in TNBC samples

(a) Distribution of the cell cycle scores of T cells (left panel) and B cells (right panel) in TNBC tumor (red) and PBMCs (blue). The cell cycle scores were calculated as the percentages of cell cycle genes' UMIs out of the total UMIs. The dotted line represents the cutoff between different proliferative statuses defined by the bimodal distribution of TNBC

T cell cycle scores (see ‘Cell cycle analysis’ in the Methods section).

(b) Distribution of the cell cycle scores of cluster 12 germinal center B cells (left panel) and the remaining (nongerminal center) B cells in TNBC tumors. The dotted line represents the cutoff between different proliferative statuses defined by the bimodal distribution of germinal center B cell cycle scores (see ‘Cell cycle analysis’ in Methods section).

(c) Percentages of proliferating cells (S and G2M) for T cells and B cells in TNBC samples and PBMC samples. The cell cycle phase was defined according to the CellCycleScoring function of the Seurat package with a modified cutoff (see ‘Cell cycle analysis’ in the Methods section).

(d) Percentages of proliferating cells (S and G2M) for C12 germinal center B cells and nongerminal center B cells. The cell cycle phase was defined according to the CellCycleScoring function of the Seurat package with a modified cutoff (see ‘Cell cycle analysis’ in the Methods section).

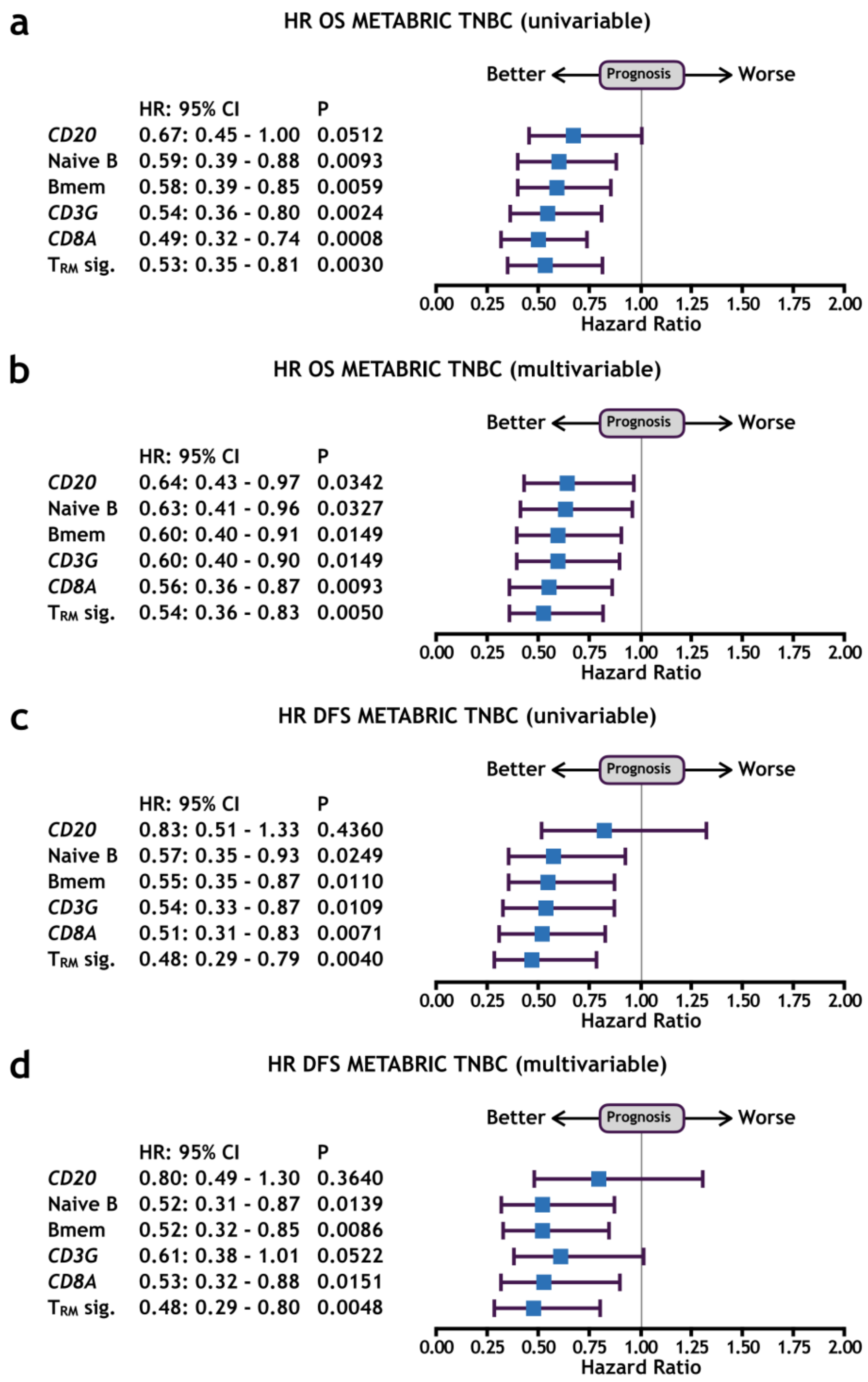

Figure S17

Supplementary Fig. 17. Survival analysis of TNBC patients in the METABRIC dataset

Prognostic effect of *CD20* and naive B cell signature, memory B cell signature, *CD3G*, *CD8A*, and tissue-resident memory T (Trm) cell signature[24]. Forest plots of hazard ratios

for univariable overall survival **(a)**, multivariable overall survival **(b)**, univariable disease-free survival **(c)**, and multivariable disease-free survival **(d)**. Forest plots show hazard ratios (the center blue squares) and 95% confidence intervals (horizontal ranges) derived from Cox regression survival analyses. Multivariable analysis was adjusted for lymph node status, tumor size, age at diagnosis, and histological grade. Data were presented as HR and 95% confidence intervals.

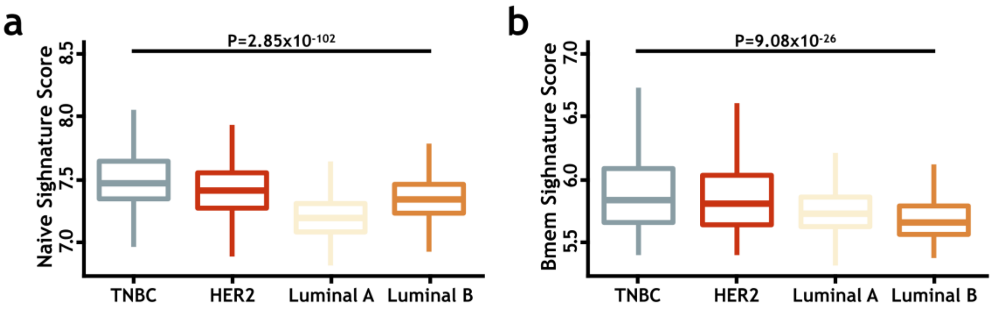

Supplementary Fig. 18. The expression levels of the B cell gene signatures in breast cancer subtypes

Box plots showing the relative enrichment of the naive B cell **(a)** and memory B cell **(b)** gene signatures in TNBC compared with other breast cancer subtypes in the METABRIC dataset. The p-values were calculated by the two-sided Kruskal–Wallis rank sum test. The breast cancer subtype classification is described in the Methods section. Box plots show the median (center bar), the third and first quartiles (upper and lower edges of the box, respectively) and the largest and smallest values that are ≤1.5 times the interquartile range (limits of the upper and lower whiskers, respectively).

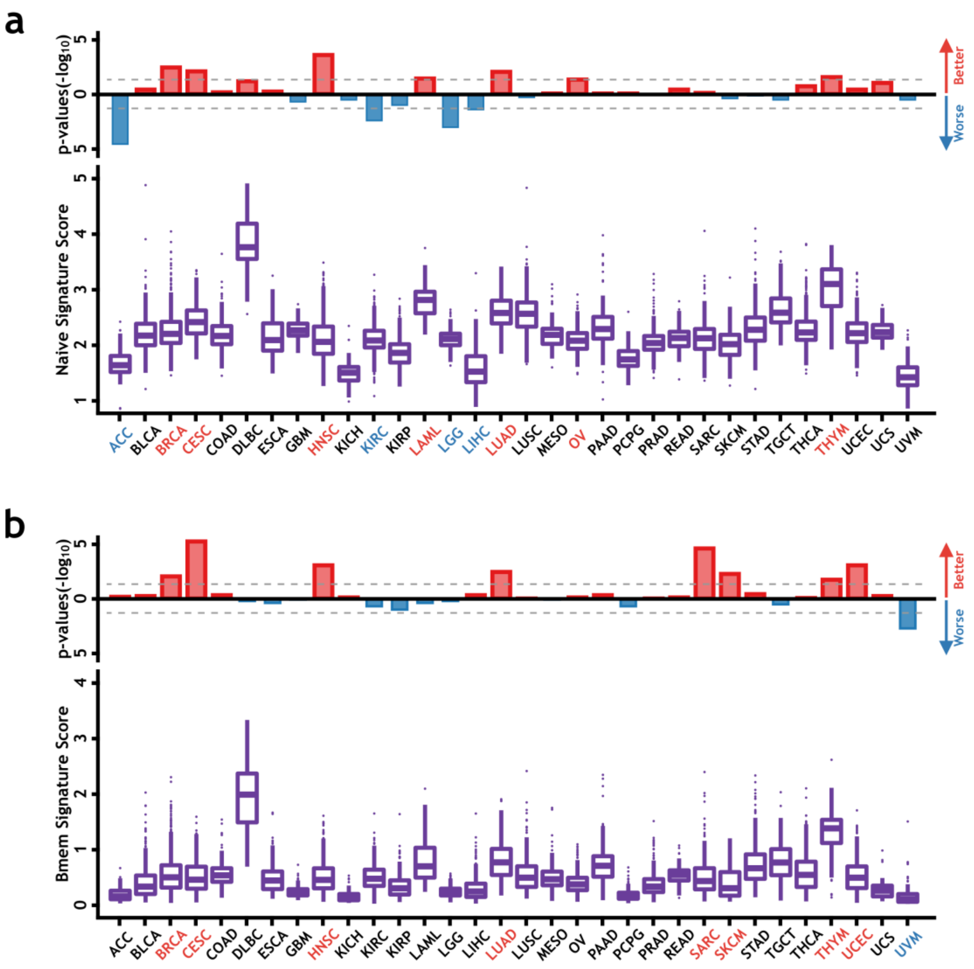

Supplementary Fig. 19. Survival analysis of different cancer types in the TCGA dataset using the naive B cell gene signature and memory B cell gene signature

The X axis represents tumor types in the TCGA dataset. The upper bar plots represent two-sided log-rank test p-values for survival analysis using the naive B cell gene signature scores **(a)** and the memory B cell gene signature scores **(b)** (higher signature score patients vs lower signature score patients). The red bars represent better survival (hazard ratios < 1), and the blue bars represent worse survival (hazard ratios > 1). The grey dotted line represents p-value=0.05. The lower panel box plots show the relative enrichment of naive B cell gene signature scores **(a)** and memory B cell gene signature scores **(b)** in each tumor type. Box plots show the median (center bar), the third and first quartiles (upper and lower edges of the box, respectively), the largest and smallest values that are ≤1.5 times the interquartile range (limits of the upper and lower whiskers, respectively), and values >1.5 times the interquartile range (dots).

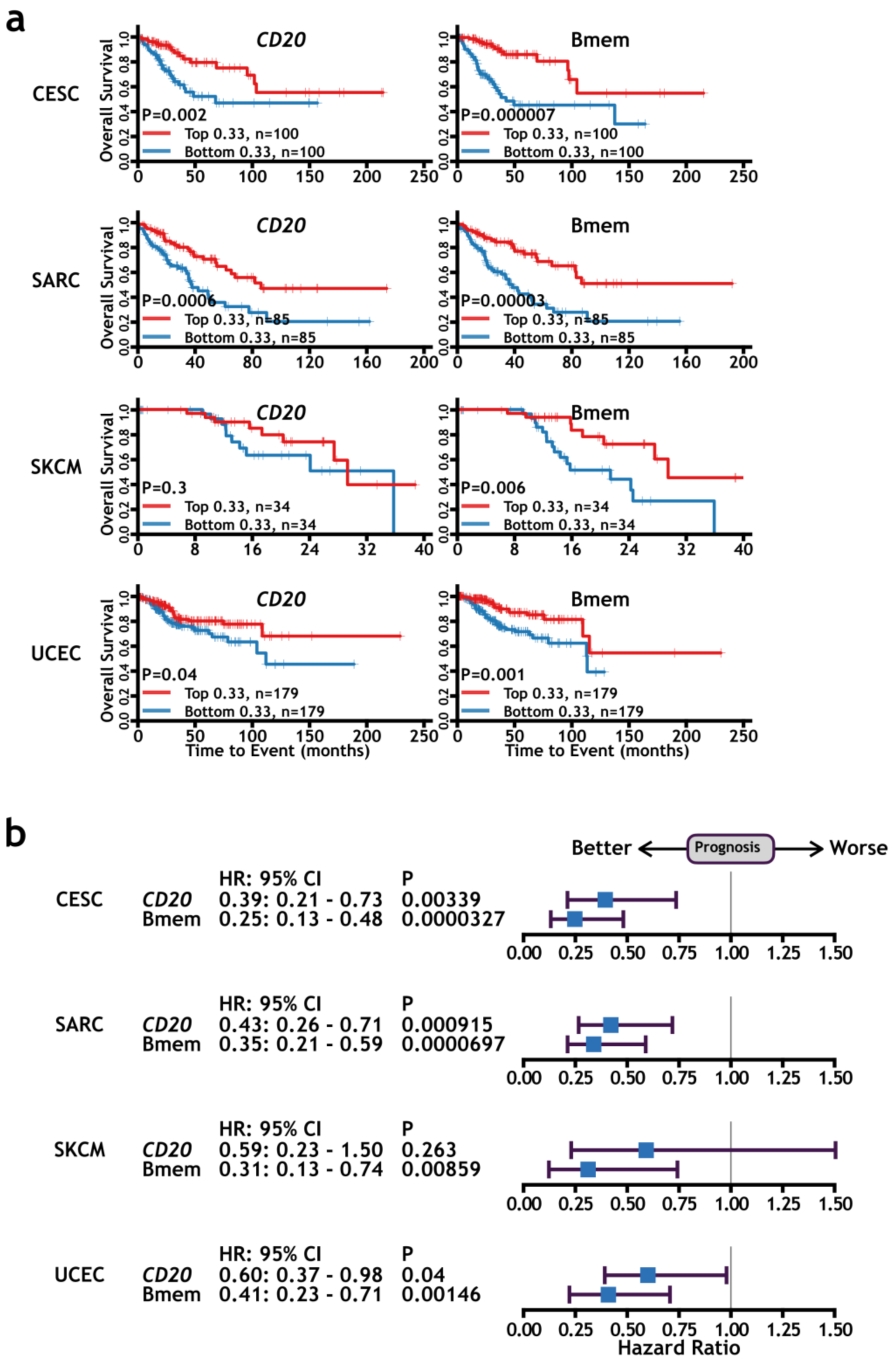

Figure S20

Supplementary Fig. 20. Survival analysis of CESC, SARC, SKCM, and UCEC samples in the TCGA dataset

(a) Kaplan–Meier survival curves for overall survival of the TCGA project (cervical squamous cell carcinoma and endocervical adenocarcinoma (CESC, n=304), sarcoma

(SARC, n=259), skin cutaneous melanoma (SKCM, n=103), and uterine corpus endometrial carcinoma (UCEC, n=543)) patients according to the expression of *CD20* and the memory B cell signature. The patients for each cancer type were sorted according to the expression levels of CD20 or the memory B cell signature, and the top 33% (n=100, 85, 34, 179 for CESC, SARC, SKCM, and UCEC) versus bottom 33% (n=100, 85, 34, 179 for CESC, SARC, SKCM, and UCEC) of patients were used to generate survival curves. The p-values were calculated by the two-sided log-rank test.

(b) Prognostic effect of *CD20* and memory B cell signature on the TCGA project (CESC, SARC, SKCM, UCEC) patients. Forest plots show HRs (the center blue squares) and 95% confidence intervals (horizontal ranges) derived from Cox regression survival analyses of overall survival in univariable analysis. Data were presented as HR and 95% confidence intervals.

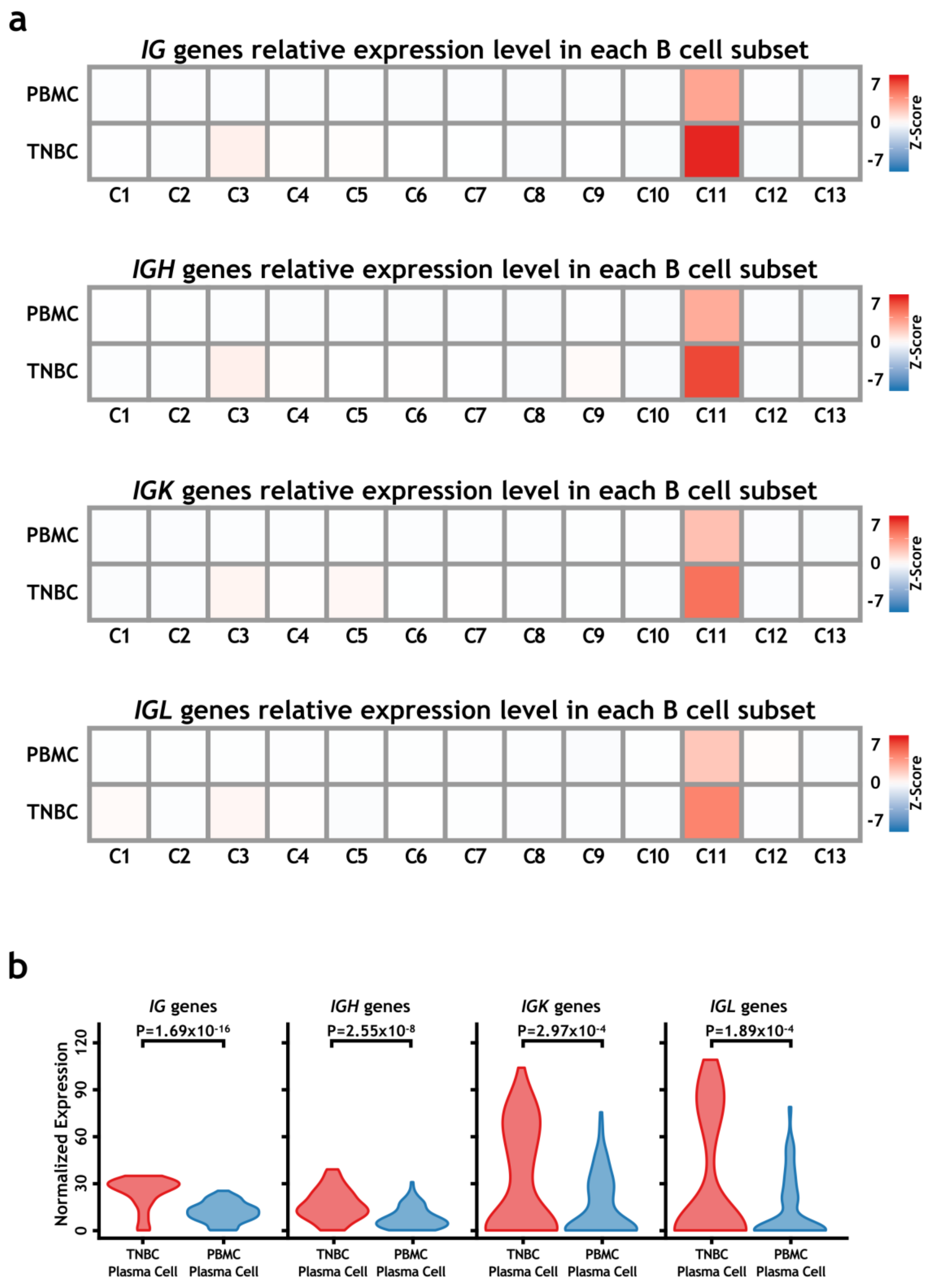

Supplementary Fig. 21. *IG* gene expression in each B cell cluster from TNBC and PBMC samples

(a) Average expression levels of total *IG*, *IGH*, *IGK*, and *IGL* genes in each B cell cluster. Upper panels represent PBMC cells; bottom panels represent TNBC cells. Each column represents one B cell cluster (C1-C13). The color scheme is based on the z-score distribution (see ‘Heatmaps of relative gene expression and signature scores for each

cluster' in the Methods section for details).

(b) The violin plots from left to right show comparisons of the average expression of total *IG*, *IGH*, *IGK*, and *IGL* genes in TNBC plasma cells (red) and PBMC plasma cells (blue). The Y axis is the sequencing depth normalized expression value. The p-values were calculated by two-tailed Student's t-test.

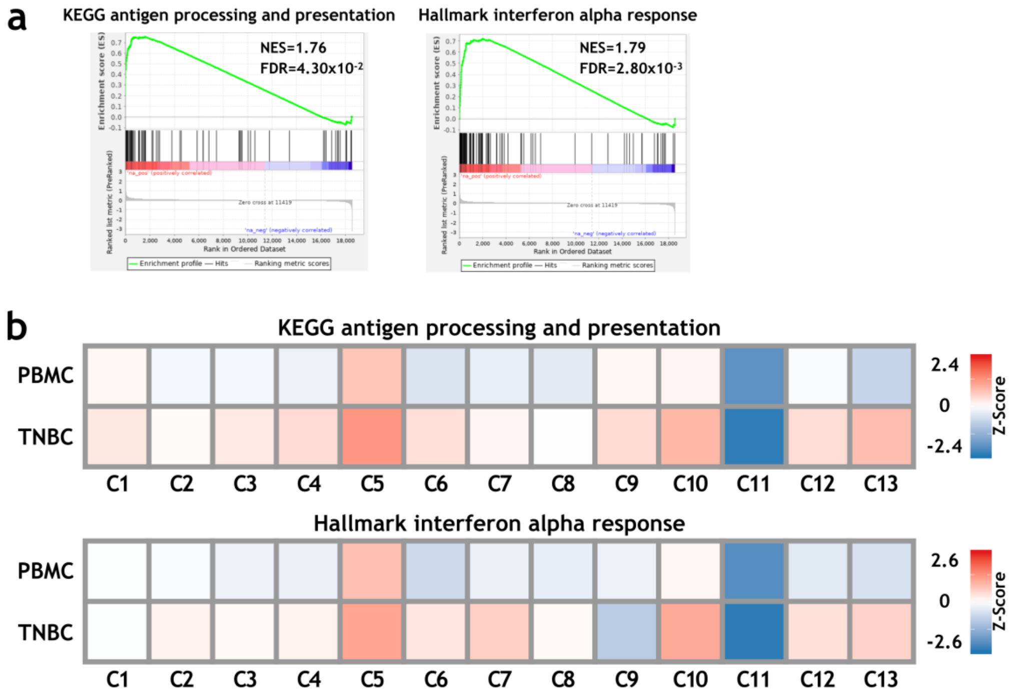

Supplementary Fig. 22. Gene set enrichment analysis (GSEA) revealed increased expression of genes involved in antigen processing and presentation and interferon alpha response genes in TNBC infiltrated B cells

(a) GSEA enrichment plots for the Kyoto Encyclopedia of Genes and Genomes (KEGG) antigen processing and presentation gene set and hallmark interferon alpha response gene set. The log fold-change ranked gene list of TNBC B cells compared to that of PBMC B cells was used for GSEA.

(b) The KEGG antigen processing and presentation gene set and hallmark interferon alpha response gene set-derived signature scores for each B cell cluster in TNBC and PBMC samples. Upper panels represent PBMC cells; bottom panels represent TNBC cells. Each column represents one B cell cluster. The color scheme is based on the z-score distribution (see ‘Heatmaps of relative gene expression and signature scores for each cluster’ in the Methods section for details).

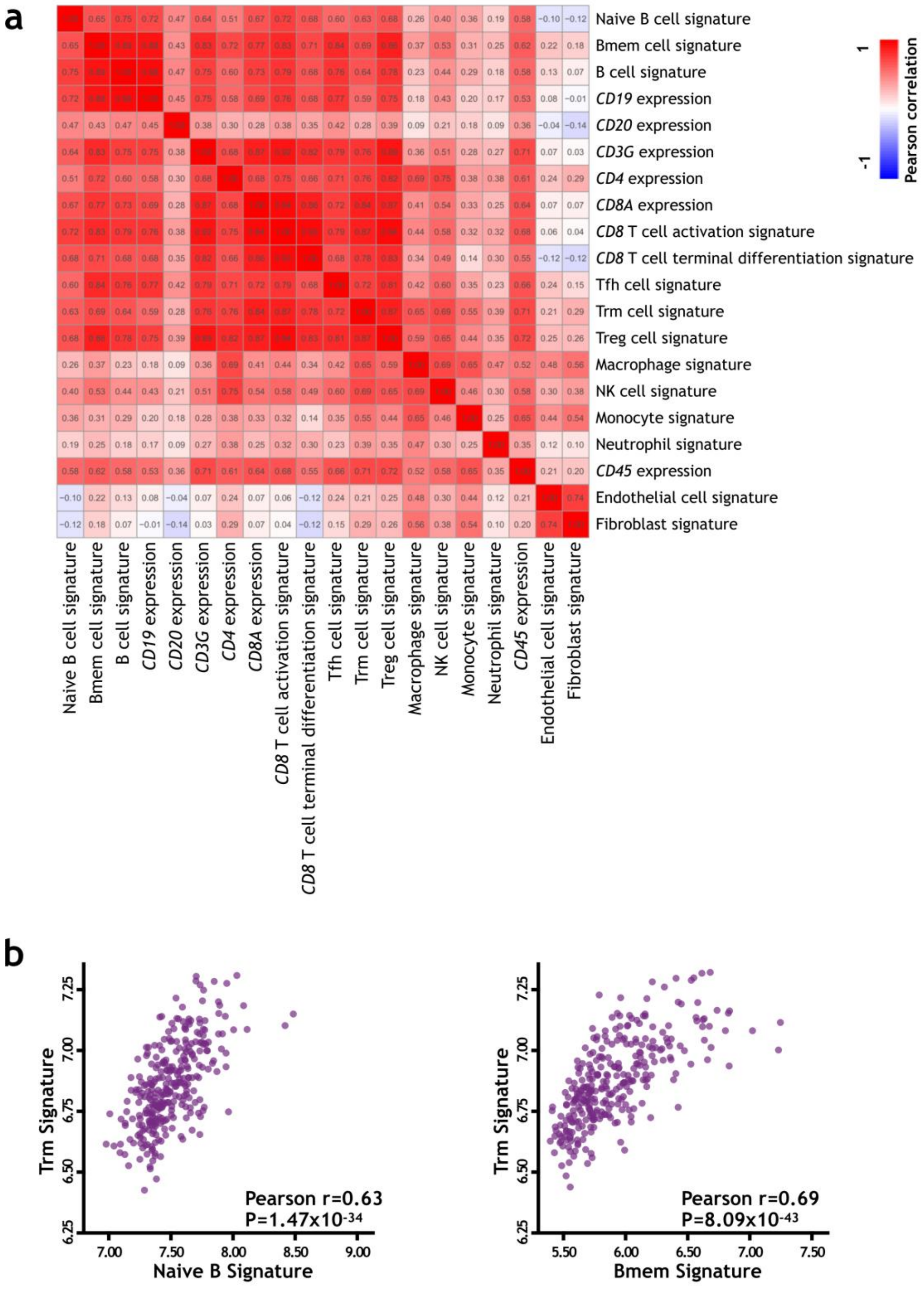

Supplementary Fig. 23. Correlations of the naive B cell and memory B cell gene signatures with other immune signatures in TNBC tumors

(a) Pearson correlation plot of the data from TNBC patients in the METABRIC dataset (n = 299). The gene names of all signatures and their sources are listed in Supplementary Table 8. Red color: positive correlation; blue color: negative correlation.

(b) Correlation plots of naive B cell signature scores (left panel) and memory B cell

signature scores (right panel) with Trm cell signature scores for each TNBC patient from the METABRIC dataset. Each dot represents one patient. The X axis and Y axis represent signature scores. Two-sided Pearson correlation values and p-values are shown in the plots.

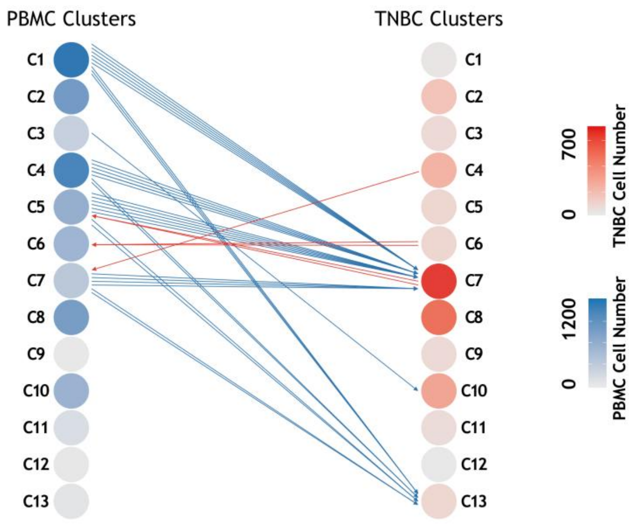

Supplementary Fig. 24. Upstream-downstream relationships between TNBC and PBMC B cells in *IGH* clonal trees

The left panel of circles (blue) represents PBMC B cell clusters, and the right panel of circles (red) represents TNBC B cell clusters. The grey to blue and grey to red color scales represent cell numbers of the clusters. Each arrow line represents the upstream-downstream relationship between one PBMC and one TNBC cell, and the cell at the arrowhead represents that it is downstream of the other cell in the *IGH* clonal tree. Blue arrow lines (n=31) represent TNBC cells downstream of PBMCs; red arrow lines (n=5) represent PBMCs downstream of TNBC cells.

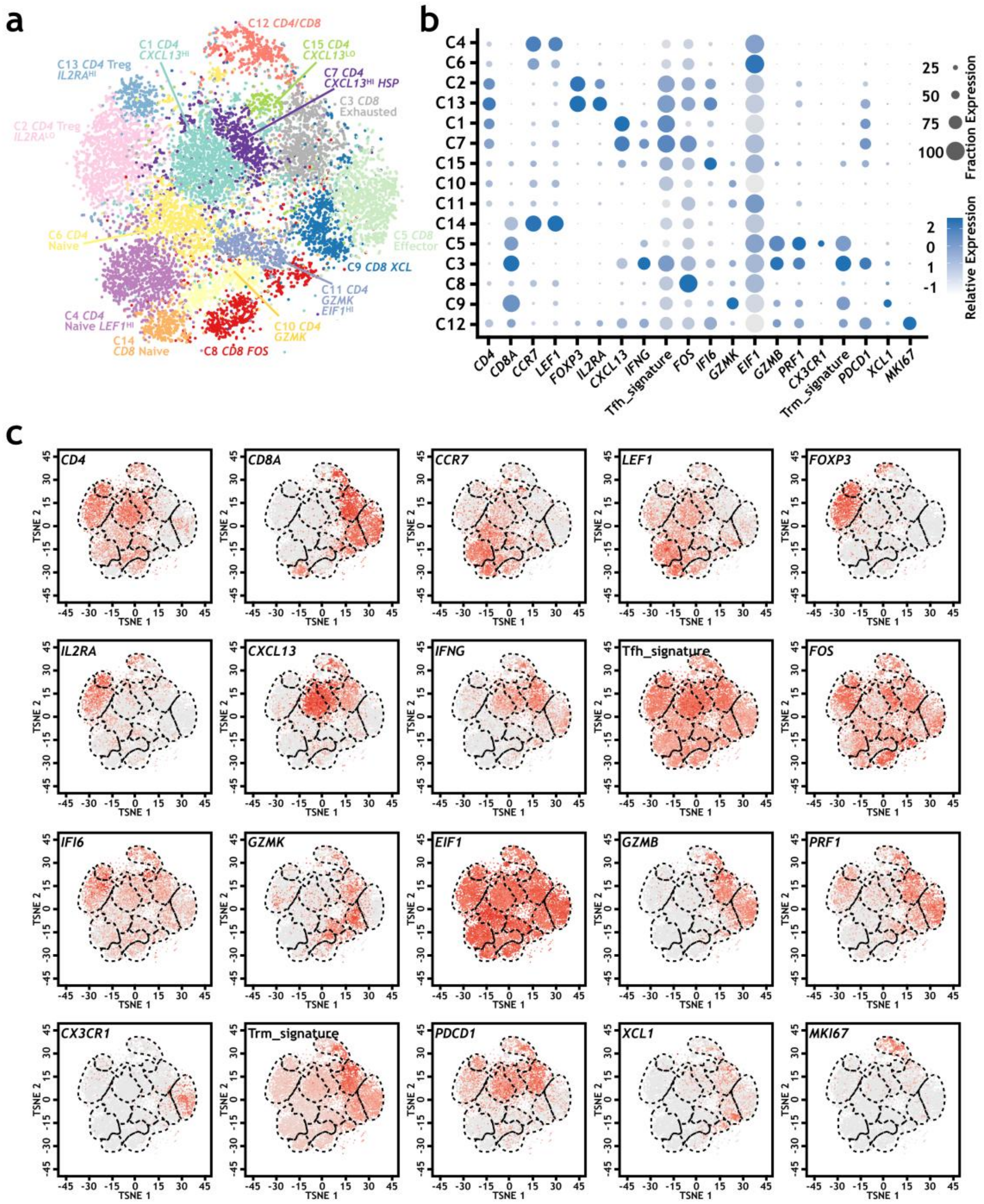

Supplementary Fig. 25. Single-cell transcriptome analysis of T cells from TNBC patients

(a) The t-SNE projection of 13,186 T cells from TNBC patients that have both RNA-seq data and a single assembled productive TCRα and TCRβ pair shows 15 major cellular clusters.

(b) Selected marker genes to define each T cell cluster.

(c) The t-SNE projections of selected marker genes in B for each T cell cluster.

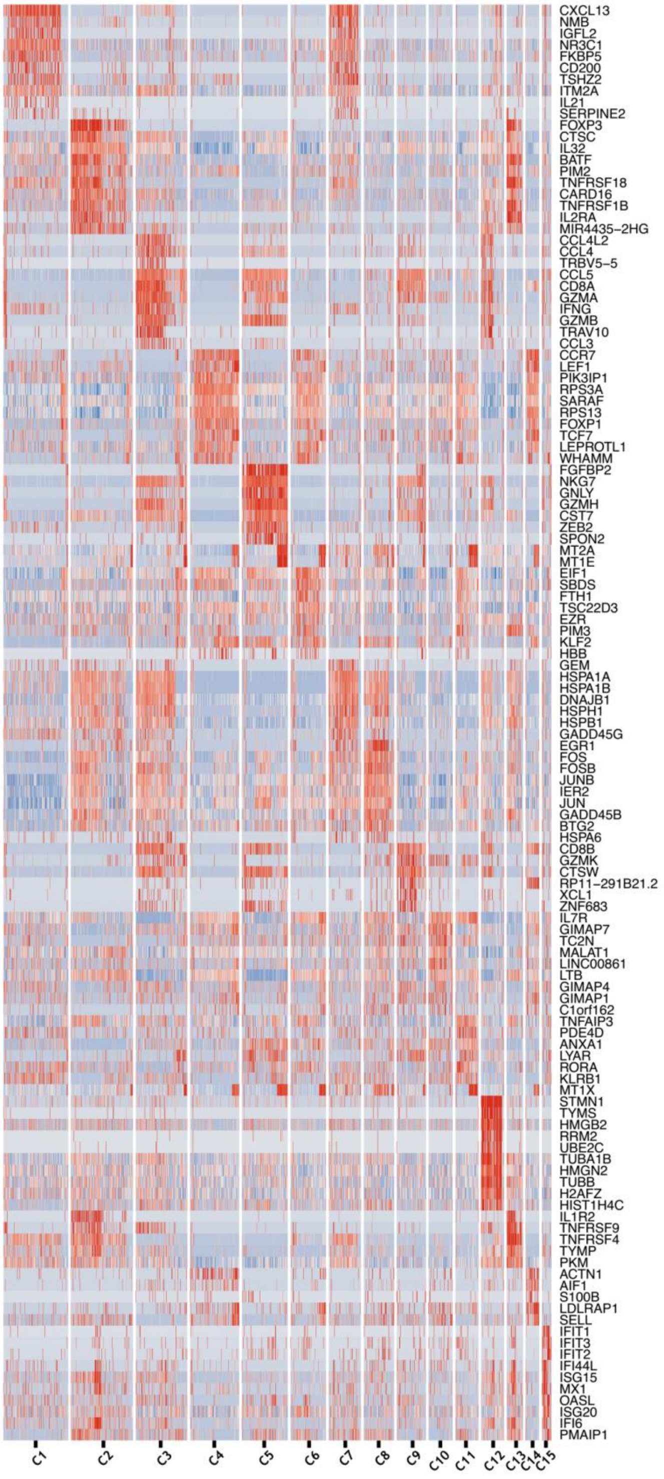

Supplementary Fig. 26. Heat map of marker genes in each T cell group

Heat map of the top 10 marker genes for the 15 T cell clusters. Gene names are labelled alongside. Red represents a high expression level, and blue represents a low expression level (also see Supplementary Data 6).

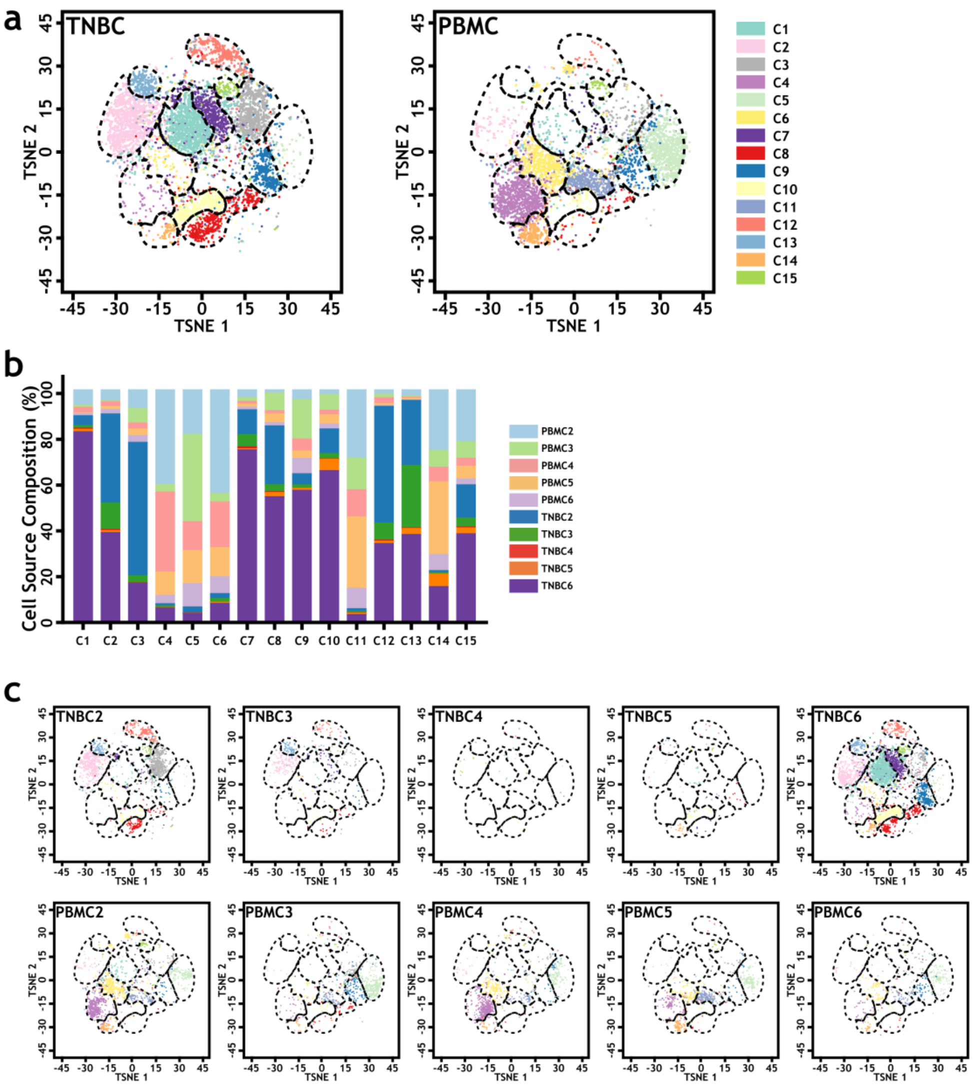

Supplementary Fig. 27. Unsupervised clustering of T cells from TNBC patients revealed 15 different cell clusters

(a) The t-SNE projections of TNBC T cells (left panel) and PBMC T cells (right panel).

(b) Percentages of cells from each sample for each T cell cluster (also see Supplementary Table 9).

(c) The t-SNE projections of T cells from each sample.

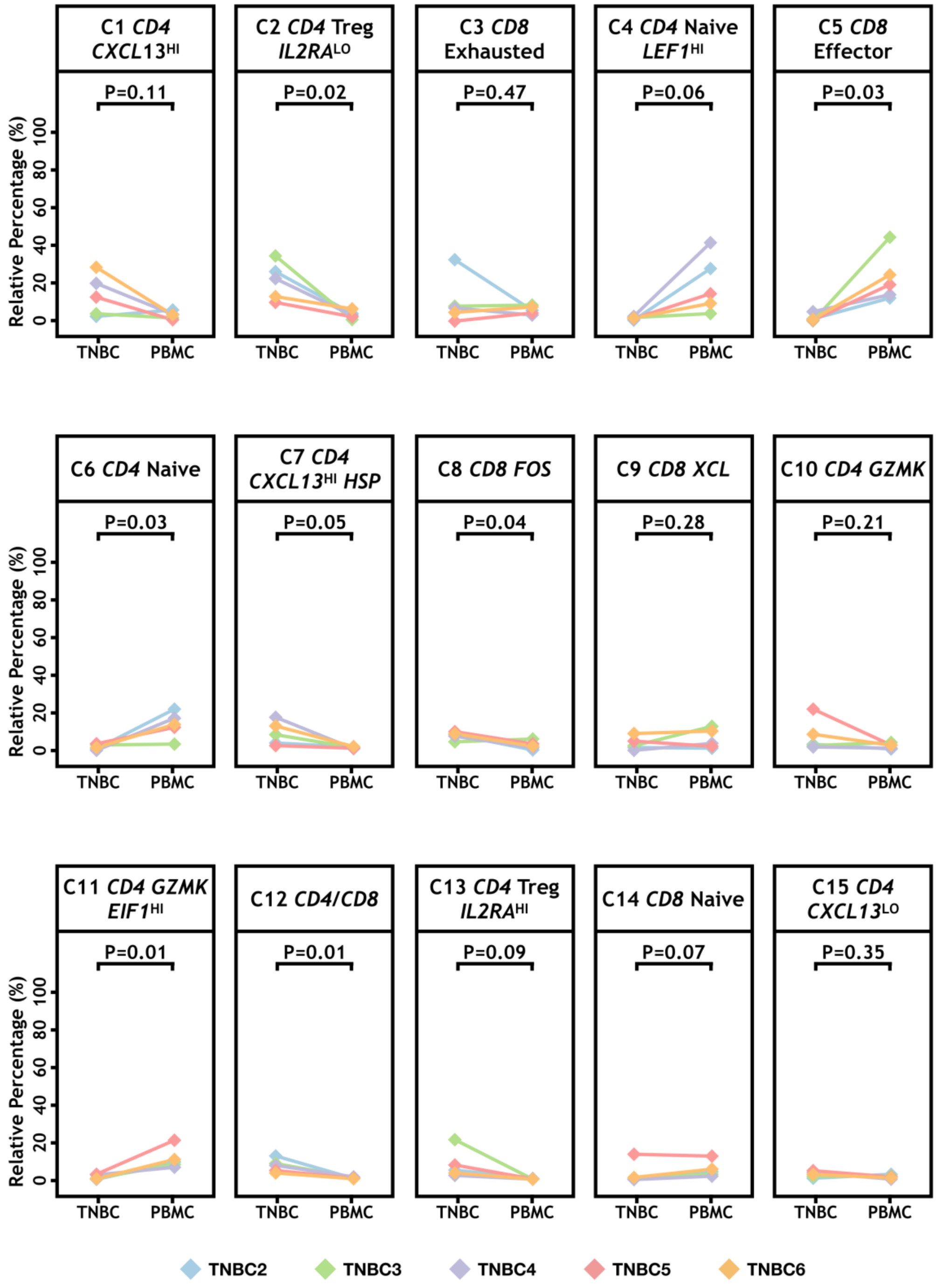

Supplementary Fig. 28. The relative percentages of different T cell clusters in TNBC and PBMC samples

The distributions of 15 clusters in each sample. The p-values were calculated by two-tailed paired Student's t-test. Source data are provided as a source data file.

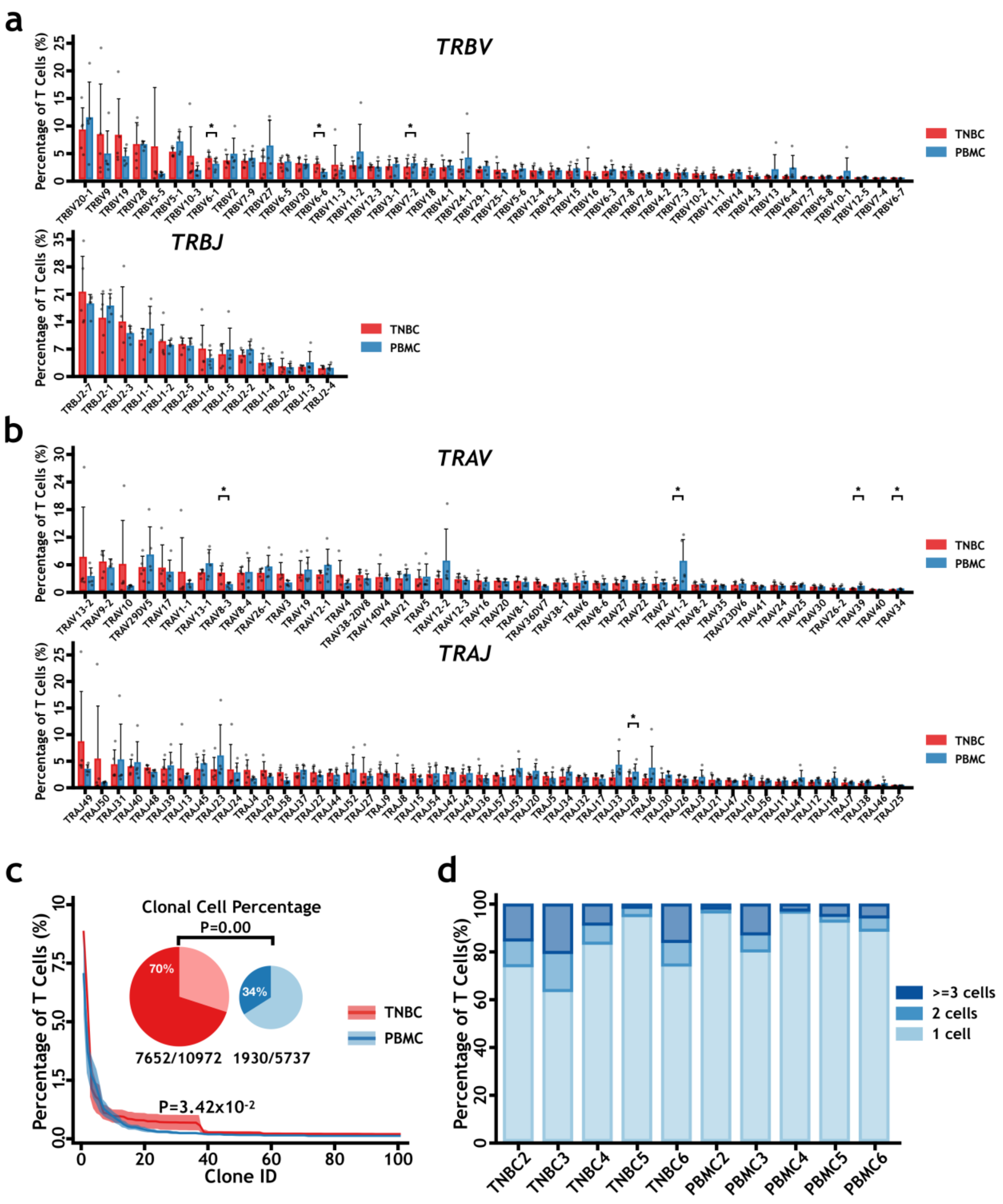

Supplementary Fig. 29. Single-cell TCR analysis in tumor and peripheral blood samples from TNBC patients

(a) Bar plots show usage of expressed V- and J- alleles of TCR beta chain in all T cells in TNBC and PBMC (a two-tailed paired Student's t-test was used, and the threshold for significance was set at $p<0.05$). Error bars represent the mean±standard deviation (SD) of the five samples. The exact p values were 0.03 (TRBV6-1), 0.03 (TRBV6-6), and 0.03 (TRBV7-2).

(b) Bar plots show usage of expressed V- and J- alleles of TCR alpha chain in all T cells in

TNBC and PBMC (a two-tailed paired Student's t-test was used, and the threshold for significance was set at $p<0.05$). Error bars represent the mean±standard deviation (SD) of the five samples. The exact p values were 0.02 (TRAV8-3), 0.05 (TRAV1-2), 0.04 (TRAV39), 0.02 (TRAV34), and 0.02 (TRAJ28).

(c) TNBC tumors contained more and larger T cell clones. The percentages of clonal T cells in all T cells are presented by the pie charts, and the p-value was calculated by two-sided Fisher's exact test. The size distribution of T cell clones shows that TNBC samples have larger T cell clones than PBMC samples. The p-value was calculated by two-tailed Student's t-test.

(d) Bar graphs show the fractions of unique and non-unique TCRs expressed in T cells from each TNBC and PBMC sample. Unique TCRs (n = 1), duplicated TCRs (n = 2), and the clonal TCRs (shared by at least 3 cells in a given cell population, n >= 3) are labeled with different colors.

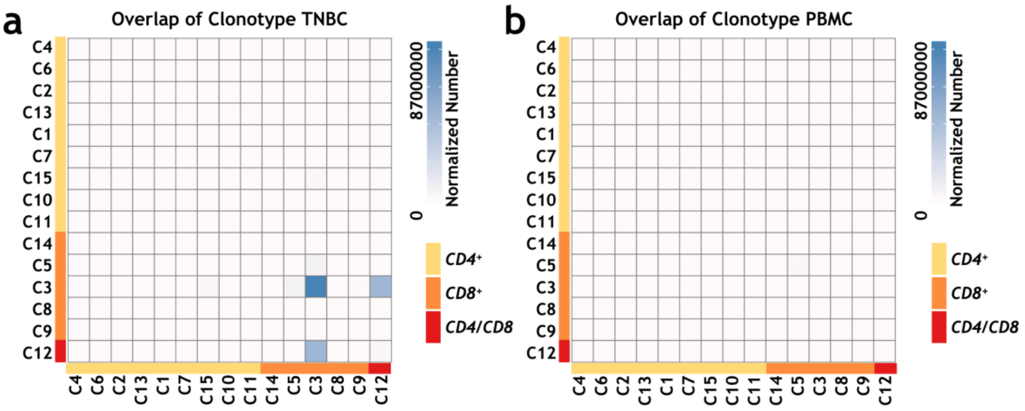

Supplementary Fig. 30. Paired TCR and single-cell RNA-seq data analyses of T cells from TNBC patients

(a) Heat map shows the distribution of the overlap of TCR clonotype among 15 T cell clusters from TNBC samples. The overlap numbers between two clusters were normalized by their cell numbers and plotted in the heatmap. See 'Paired TCR and single-cell RNA-seq data analyses' in the Supplementary Methods section for details.

(b) Heat map shows the distribution of the overlap of TCR clonotype among 15 T cell clusters from PBMC samples. The overlap numbers between two clusters were normalized by their cell numbers and plotted in the heatmap. See 'Paired TCR and single-cell RNA-seq data analyses' in the Supplementary Methods section for details.

Supplementary Table 1. Clinical information of patients

Patient ID	Experiment	Subtype	Age	Gender	Time of Clinical diagnosis	Time of Sample collection during surgery	Time of Pathological report	SBR grade	ER (IHC)	PR (IHC)	HER-2 (IHC)	HER-2 (FISH)	Ki-67 (IHC)	Histology of Primary Tumor	Axillary Node Status
TNBC1	FACS,scRNA	TNBC	54	Female	2018/2/23	2018/2/27	2018/3/1	III	-	-	1+	NA	70%	ductal	-
TNBC2	FACS,scRNA	TNBC	51	Female	2018/2/23	2018/3/13	2018/3/18	II-III	-	-	-	NA	30%	others	-
TNBC3	FACS,scRNA	TNBC	47	Female	2018/4/13	2018/4/19	2018/4/24	II	-	-	1+	NA	50%	ductal	-
TNBC4	FACS,scRNA	TNBC	78	Female	2018/5/3	2018/5/7	2018/5/11	II	-	-	2+	-	30%	ductal	1/10
TNBC5	FACS,scRNA	TNBC	53	Female	2018/5/8	2018/5/14	2018/5/19	II	-	-	-	NA	70%	ductal	-
TNBC6	FACS,scRNA	TNBC	63	Female	2018/5/10	2018/5/14	2018/5/18	II	-	-	1+	NA	40%	ductal	1/3
TNBC7	FACS	TNBC	68	Female	2018/9/10	2018/9/13	2018/9/18	II	-	-	-	NA	40%	others	-
TNBC8	FACS	TNBC	63	Female	2017/12/18	2017/12/26	2018/1/2	III	-	-	-	NA	70%	ductal	17/22
TNBC9	FACS	TNBC	61	Female	2017/10/30	2017/11/1	2017/11/7	II-III	-	-	2+	NA	30%	ductal	0/4
TNBC10	FACS	TNBC	55	Female	2017/9/14	2017/9/17	2017/9/21	II	-	-	1+	NA	50%	ductal	-
TNBC11	FACS	TNBC	54	Female	2017/12/26	2017/12/26	2017/12/30	II	90+	80%	3+	NA	60%	ductal	-
TNBC12	FACS	TNBC	50	Female	2017/12/18	2017/12/29	2018/1/4	II	-	-	1+	NA	80	ductal	-
TNBC13	FACS	TNBC	41	Female	2018/8/13	2018/8/15	2018/8/23	II	-	-	1+	NA	30%	ductal	5/20
TNBC14	FACS	TNBC	55	Female	2018/1/29	2018/1/31	2018/2/4	II	-	-	+	NA	40%	ductal	-
TNBC15	FACS	TNBC	55	Female	2018/3/29	2018/4/2	2018/4/10	II	-	-	-	NA	95%	ductal	-
TNBC16	FACS	TNBC	43	Female	2018/9/11	2018/9/13	2018/9/20	II	-	-	1+	NA	60%	ductal	3/19
TNBC17	FACS	TNBC	45	Female	2017/12/23	2017/12/27	2018/1/5	II	-	-	1+	NA	20%	ductal	-
LBBC1	FACS	Luminal B	73	Female	2018/9/11	2018/9/13	2018/9/18	II	50%	1%	1+	NA	80%	ductal	-
LBBC2	FACS	Luminal B	61	Female	2018/4/22	2018/5/14	2018/5/19	II	-	90%	-	NA	80%	ductal	-
LBBC3	FACS	Luminal B	60	Female	2018/6/15	2018/6/21	2018/6/27	I	90%	-	-	NA	70%	ductal	4/14
LBBC4	FACS	Luminal B	54	Female	2018/9/7	2018/9/11	2018/9/15	II	-	-	1+	NA	70%	ductal	-
LBBC5	FACS	Luminal B	49	Female	2018/4/23	2018/4/25	2018/4/29	II	50%	1%	1+	NA	80%	ductal	-
LBBC6	FACS	Luminal B	42	Female	2018/9/4	2018/9/6	2018/9/11	I	40%	70%	3+	NA	20%	ductal	-
LABC1	FACS	Luminal A	75	Female	2018/8/20	2018/8/23	2018/8/27	II	95%	70%	2+	-	30%	ductal	5/10
LABC2	FACS	Luminal A	66	Female	2018/8/10	2018/8/17	2018/8/21	III	95%	80%	2+	-	10%	ductal	-
LABC3	FACS	Luminal A	64	Female	2018/5/15	2018/5/18	2018/5/23	II	90%	70%	2+	-	30%	ductal	-
LABC4	FACS	Luminal A	62	Female	2018/6/13	2018/6/19	2018/6/26	II	90%	90%	1+	NA	20%	ductal	-
LABC5	FACS	Luminal A	61	Female	2018/4/23	2018/4/25	2018/4/29	II	90%	5%	1+	NA	15%	ductal	8/23
LABC6	FACS	Luminal A	60	Female	2018/5/3	2018/5/10	2018/5/14	II	80%	5%	2+	-	25%	ductal	2/14
LABC7	FACS,scRNA	Luminal A	56	Female	2018/5/11	2018/5/14	2018/5/18	II	90%	40%	2+	-	20%	ductal	-
LABC8	FACS,scRNA	Luminal A	53	Female	2018/8/14	2018/8/22	2018/8/26	I	90%	50%	-	NA	10%	ductal	-
LABC9	FACS,scRNA	Luminal A	57	Female	2018/9/5	2018/9/11	2018/9/16	II	80%	80%	+	NA	10%	ductal	4/14
LABC10	FACS	Luminal A	53	Female	2017/10/27	2017/10/30	2017/11/3	II	90%	20%	1+	NA	10%	ductal	1/14
LABC11	FACS	Luminal A	51	Female	2018/5/9	2018/5/14	2018/5/19	III	40%	-	-	NA	10%	ductal	-
LABC12	FACS	Luminal A	46	Female	2018/9/9	2018/9/12	2018/9/17	II-III	60%	90%	1+	NA	40%	ductal	-
LABC13	FACS	Luminal A	45	Female	2018/4/19	2018/4/23	2018/4/27	II	90%	70%	2+	-	25%	ductal	-
LABC14	FACS	Luminal A	32	Female	2017/10/27	2017/10/30	2017/11/4	II	90%	90%	1+	NA	20%	ductal	-
HER2BC1	FACS	HER2	55	Female	2017/12/4	2017/12/7	2017/12/14	II	-	-	3+	NA	70%	ductal	-
HER2BC2	FACS	HER2	55	Female	2018/5/10	2018/5/14	2018/5/18	II	-	-	3+	NA	20%	ductal	3/15
HER2BC3	FACS	HER2	47	Female	2018/9/18	2018/9/20	2018/9/25	II	-	-	3+	NA	70%	ductal	1/26
HER2BC4	FACS	HER2	37	Female	2017/12/20	2017/12/22	2017/12/28	II-III	5%	5%	3+	NA	60%	ductal	-
HER2BC5	FACS	HER2	66	Female	2018/4/30	2018/5/2	2019/5/8	III	90%	-	3+	NA	70%	ductal	5/21
HER2BC6	FACS	HER2	49	Female	2018/3/8	2018/3/17	2018/3/24	II	-	-	3+	NA	20%	ductal	-
HER2BC7	FACS	HER2	55	Female	2018/5/4	2018/5/7	2018/5/11	II	90+	10%	3+	NA	50%	ductal	6/7
HER2BC8	FACS	HER2	45	Female	2018/3/5	2018/3/12	2018/3/16	II	-	-	3+	NA	60%	ductal	3/13
HER2BC9	FACS	HER2	45	Female	2018/4/26	2018/5/3	2018/5/8	II	95%	95%	2+	NA	40%	ductal	7/17
HER2BC10	FACS,scRNA	HER2	51	Female	2018/8/20	2018/8/23	2018/8/31	II-III	-	-	3+	NA	50%	ductal	-
HER2BC11	FACS	HER2	49	Female	2018/1/2	2018/1/6	2018/1/10	II	-	-	3+	NA	25%	ductal	-
HER2BC12	FACS	HER2	48	Female	2018/1/22	2018/1/24	2018/1/30	II-III	-	-	3+	NA	50%	ductal	19/23
HER2BC13	FACS	HER2	78	Female	2018/6/12	2018/6/21	2018/6/25	III	-	-	3+	+	30%	ductal	12/14
HER2BC14	FACS	HER2	55	Female	2017/8/18	2017/11/30	2017/8/2	II	-	-	3+	NA	10%	ductal	1/8
HER2BC15	FACS	HER2	65	Female	2018/4/10	2018/4/13	2018/4/17	II	80%	80%	2+	+	20%	ductal	-
HER2BC16	FACS	HER2	67	Female	2018/3/28	2018/4/4	2018/4/9	II	-	-	3+	NA	40%	ductal	5/12

Supplementary Table 2. Statistic information of the scRNA-seq libraries

			Before cell filtration [*]					Number of cell after filtration		After cell filtration		
Sample_ID	Tissue	10x chemistry	Detected cell number	Mean reads per cell	Sequencing saturation	Median genes per cell	Median UMI counts per cell	Number of cell after filtering doublet[&]	Number of cell after filtering low quality cells[#]	Median genes per cell	Median UMI counts per cell	Median mitochondrial RNA fraction
PBMC1	Blood	Single cell 3' v2	15408	41016	84.30%	1034	2702	13214	13180	1012	2605	2.43%
PBMC2	Blood	Single cell 5' VDJ	7997	40341	88.60%	1522	4858	7379	7205	1520	4850	2.75%
PBMC3	Blood	Single cell 5' VDJ	3662	85267	94.40%	1490	4193	3518	3507	1486	4184	4.42%
PBMC4	Blood	Single cell 5' VDJ	7002	68615	91.80%	1594	5438	6522	6252	1603	5416	5.48%
PBMC5	Blood	Single cell 5' VDJ	5914	191921	94.70%	2224	7706	5566	5385	2165	7391	4.16%
PBMC6	Blood	Single cell 5' VDJ	5445	145649	93.30%	2240	7490	5146	4904	2179	7169	4.49%
PBMC7	Blood	Single cell 5' VDJ	8172	59603	87.10%	2040	6152	7528	7457	1991	5930	3.80%
PBMC8	Blood	Single cell 5' VDJ	4116	65853	91.60%	1050	2401	3939	3782	1078	2442	6.79%
PBMC9	Blood	Single cell 5' VDJ	9929	64889	90.50%	1817	5528	8993	8920	1794	5472	3.60%
PBMC10	Blood	Single cell 5' VDJ	8588	72761	92.20%	1785	5450	7880	7849	1766	5358	3.31%
TNBC1	Tumor	Single cell 3' v2	6088	61598	80.50%	1169	3411	5720	5646	1151	3354	1.34%
TNBC2	Tumor	Single cell 5' VDJ	7969	50713	80.20%	2406	7747	7350	7111	2267	6991	3.27%
TNBC3	Tumor	Single cell 5' VDJ	3778	54503	82.60%	1908	5736	3626	3590	1866	5545	2.81%
TNBC4	Tumor	Single cell 5' VDJ	1200	124933	92.10%	1591	5914	1179	1060	1511	5482	0.21%
TNBC5	Tumor	Single cell 5' VDJ	2127	80711	87.10%	1773	5573	2072	2015	1754	5469	3.36%
TNBC6	Tumor	Single cell 5' VDJ	13070	59257	88.80%	1710	4462	11477	11362	1649	4243	2.67%
LABC7	Tumor	Single cell 5' VDJ	739	46283	86.90%	1340	3357	730	710	1336	3341	3.93%
LABC8	Tumor	Single cell 5' VDJ	4475	45407	85.20%	1259	3676	4268	4241	1241	3609	3.61%
LABC9	Tumor	Single cell 5' VDJ	4209	80800	88.90%	1213	3171	4024	3996	1203	3138	5.61%
HER2BC10	Tumor	Single cell 5' VDJ	5041	43096	84.80%	1468	4327	4783	4766	1439	4234	3.75%

[*] From 10x Cell Ranger output

[&] See method part for doublet removement

[#] Low quality cells are defined as cells with number of expressed gene < 200, > 5000, or with mitochondiral RNA UMI percentage > 50%

Supplementary Table 3. Statistic information of the BCR and TCR libraries

				BCR library*				IMGT/HighV-QUEST annotation	TCR library *		
**Sample ID**	**Tissue**	**10x chemistry**	**Single cell RNA-seq library detected**	**Mean read pairs per Cell**	**Estimate number of cells**	**Cell count confidence**	**Cells with IGH contig**	**Cells with single productive IgH**	**Mean read pairs per cell**	**Estimate number of cells**	**Cell count confidence**
PBMC2	Blood	Single cell 5' VDJ	7997	18.2k	358	98.10%	325	267	3.6k	4736	85.90%
PBMC3	Blood	Single cell 5' VDJ	3662	32.4k	526	98.90%	444	365	9k	2404	91.30%
PBMC4	Blood	Single cell 5' VDJ	7002	5.3k	1386	97.30%	1195	671	4.0k	4127	90.50%
PBMC5	Blood	Single cell 5' VDJ	5914	11.6k	5102	97.90%	4878	3709	5.0k	2331	81.80%
PBMC6	Blood	Single cell 5' VDJ	5445	13.8k	4169	98.20%	4019	3025	4.2k	1644	87.20%
PBMC7	Blood	Single cell 5' VDJ	8172	6k	5458	98.10%	5038	3764	5.2k	3538	72.40%
PBMC8	Blood	Single cell 5' VDJ	4116	6.2k	3093	92.80%	2883	2710	13.1K	361	52.70%
PBMC9	Blood	Single cell 5' VDJ	9929	5.1k	2030	93.90%	1874	1585	6.4k	7711	92.30%
PBMC10	Blood	Single cell 5' VDJ	8588	10.8K	536	99.40%	478	389	5.4k	5902	83.70%
TNBC2	Tumor	Single cell 5' VDJ	7969	20.6k	1087	98.90%	999	815	4.7k	5571	92.80%
TNBC3	Tumor	Single cell 5' VDJ	3778	15.4k	612	96.90%	437	221	23.0k	1187	84.70%
TNBC4	Tumor	Single cell 5' VDJ	1200	36.7k	222	95.00%	178	129	70.4k	104	91.60%
TNBC5	Tumor	Single cell 5' VDJ	2127	6.5k	1974	96.10%	1729	1261	10.0k	816	83.70%
TNBC6	Tumor	Single cell 5' VDJ	13070	4.5k	3513	91.60%	2579	1269	4.9k	9895	86.50%
LABC7	Tumor	Single cell 5' VDJ	739	21.1K	198	97.00%	171	157	56.3k	72	100.00%
LABC8	Tumor	Single cell 5' VDJ	4475	5.4k	1600	93.70%	1334	1022	6.2k	2478	84.50%
LABC9	Tumor	Single cell 5' VDJ	4209	6.6k	1300	91.30%	1105	905	6.1k	1798	89.30%
HER2BC10	Tumor	Single cell 5' VDJ	5041	10.0k	345	97.10%	242	172	4.8k	3420	86.70%

*From 10x Cell Ranger default output

Supplementary Table 4. Cell numbers of 4 CD45+ cell clusters according to sample of origin

	TNBC1	TNBC2	TNBC3	TNBC4	TNBC5	TNBC6	LABC7	LABC8	LABC9	HER2BC10	PBMC1	PBMC2	PBMC3	PBMC4	PBMC5	PBMC6	PBMC7	PBMC8	PBMC9	PBMC10
T cells	3213	3104	907	227	624	7553	197	2356	3512	2398	7329	5985	2390	4100	1324	2063	2101	645	5636	6555
B cells	1371	651	424	113	1018	2266	202	1341	221	1210	608	358	494	1138	3073	1932	3822	2672	420	1350
NK cells	190	159	33	19	10	563	9	99	238	144	3704	524	282	494	235	189	595	65	1574	988
macrophage/monocyte/neutrophil	872	3197	2226	701	363	980	302	445	795	244	1539	338	341	520	753	720	939	400	219	27

Supplementary Table 5. Cell numbers of 4 B cell clusters according to sample of origin

	TNBC2	TNBC3	TNBC4	TNBC5	TNBC6	PBMC2	PBMC3	PBMC4	PBMC5	PBMC6
Naive	49	19	4	98	94	121	70	377	704	945
Bmem	406	141	62	750	725	104	260	202	1572	1608
Plasma	6	15	18	12	25	6	11	24	36	27
$CD14^+$ B	32	10	4	40	16	2	1	1	15	20

Supplementary Table 6. Cell numbers of 13 B cell clusters according to sample of origin

	TNBC2	TNBC3	TNBC4	TNBC5	TNBC6	PBMC2	PBMC3	PBMC4	PBMC5	PBMC6
C1 *NUR77* ^{High} Naive	14	4	1	11	16	37	28	88	437	595
C2 Naive B	25	4	1	81	62	78	27	232	175	279
C3 *CCR7* ^{High} Bmem	25	20	2	15	32	8	15	56	97	67
C4 *IGM* $^{+}$ *CD27* $^{+}$ Bmem	73	9	14	56	87	25	52	38	419	490
C5 *CD27* $^{-}$ Atypical Bmem	10	3	2	16	73	10	51	23	161	329
C6 *CD1C* ^{High} Bmem	17	19	2	26	40	21	50	21	226	198
C7 *CD24* ^{High} Bmem	57	24	22	253	335	14	21	36	141	109
C8 Switched Bmem	157	5	3	246	60	20	40	52	336	314
C9 *NUR77* ^{high} Bmem	3	3	5	76	4	0	0	0	1	0
C10 *CD99* ^{high} Bmem	67	66	9	50	97	13	45	31	282	167
C11 Plasma	6	17	21	12	27	5	11	24	36	28
C12 Germinal center B	6	0	2	17	11	0	1	2	1	3
C13 *CD14* $^{+}$ B	33	11	4	41	16	2	1	1	15	21

Supplementary Table 7. Signature genes of the B cell groups

Naive B	logfc	pvalue	stat	fdr
STMN1	1.353223	4.64E-16	8.120511	2.07E-14
MARCKSL1	1.4004	1.33E-60	16.42221	7.06E-58
RGS1	1.063592	2.60E-10	6.321193	4.87E-09
RFTN1	1.344032	2.04E-21	9.502969	1.59E-19
CD38	1.667075	6.57E-12	6.866722	1.64E-10
HMGB2	1.120897	1.70E-22	9.758551	1.48E-20
SSBP2	1.112365	1.00E-10	6.466578	2.03E-09
PTTG1	1.181657	9.20E-12	6.818537	2.24E-10
BACH2	1.709291	2.70E-38	12.93929	6.88E-36
STAG3	1.24081	8.59E-17	8.322867	4.30E-15
PLPP5	1.224624	5.00E-26	10.5515	5.97E-24
CD72	1.312224	7.26E-51	15.00074	3.04E-48
LRMP	1.173689	3.50E-31	11.61405	5.69E-29
SYNE2	1.018025	6.06E-10	6.188988	1.02E-08
IGHD	1.983171	2.25E-31	11.65152	4.00E-29
GCHFR	1.069663	6.98E-09	5.791415	9.37E-08
ELL3	1.825134	4.46E-30	11.39448	6.69E-28
NEIL1	1.589287	6.27E-22	9.625025	5.10E-20
MCTP2	1.003631	1.05E-09	6.101391	1.71E-08
RP11-231C	1.52689	5.96E-32	11.76431	1.09E-29
DEF8	1.552159	4.89E-20	9.166434	3.33E-18
PRPSAP2	1.135778	6.89E-20	9.129302	4.59E-18
FCER2	1.702499	7.90E-51	14.99518	3.08E-48

Bmem	logfc	pvalue	stat	fdr
CD1C	1.046373	4.21E-19	8.931413	9.19E-18
ADAMTS6	1.063102	1.44E-08	5.668261	1.05E-07
CXCR3	1.135169	9.49E-14	7.447792	1.33E-12
LCN10	1.162353	9.08E-09	5.747065	6.77E-08
CTD-2509(	1.379396	9.43E-12	6.81493	1.08E-10
TNFRSF13I	1.143594	4.24E-51	15.03639	5.52E-49
ZBTB32	1.445812	3.90E-15	7.858013	6.14E-14

C2 Naive B	logfc	pvalue	stat	fdr
C1orf162	1.451819	0.000166	3.766553	0.00086281
BACH2	1.503255	1.69E-14	7.672555	8.98E-13
PLPP5	1.379486	1.72E-20	9.278314	1.80E-18
CD72	1.358441	6.80E-26	10.5225	9.96E-24
YBX3	1.017439	2.08E-05	4.25666	0.000144267
SLC16A7	1.122375	4.88E-07	5.030921	5.43E-06
IGHD	1.727505	2.19E-15	7.930359	1.24E-13
ELL3	1.017272	2.47E-05	4.217855	0.000168473
RP11-231C	1.236576	2.03E-10	6.359179	5.32E-09
C16orf74	1.00007	1.22E-06	4.851818	1.23E-05
FCER2	1.546679	1.86E-25	10.42758	2.53E-23
IGLL5	1.366856	1.37E-08	5.676904	2.25E-07

The statistical test used was two-sided likelihood-ratio test, and adjustments were made for multiple comparisons in the fdr column

Supplementary Table 8. Signature genes for correlation analysis

Naive B cell signature (this study)	Bmem cell signature (this study)	B cell signature[7]	CD8 T cell activation[8]	CD8 T cell terminal differentiation signature[8]	Tfh cell signature[9]	Trm cell signature[4]	Treg cell signature[9]	Macrophage signature[9]	NK cell signature[9]	Monocyte signature[9]	Neutrophil signature[9]	Endothelial cell signature[10]	Fibroblast signature[10]
STMN1	CD1C	IGJ	CD69	TIGIT	B3GAT1	NBL1	NT5E	AIF1	AKT3	ACTR3	ABTB1	PECAM1	FAP
MARCKSL1	ADAMTS6	KIAA0125	CCR7	PDCD1	BCL6	RP4-728D4.2	CD3D	CCL1	AXL	ANXA5	AMPD2	VWF	THY1
RGS1	CXCR3	TNFRSF17	CD27	CD274	CCR7	LMO4	CD3G	CCL14	CDH2	ARPC2	CAMP	CDH5	DCN
RFTN1	LCN10	FCRL5	BTLA	CTLA4	CD200	VCAM1	CD3E	CCL26	CRTAM	ATP6V1B2	EMR4P	CLDN5	COL1A1
CD38	CTD-2509G16.5	POU2AF1	CD40LG	LAG3	CD83	MLLT11	CD4	CD163	CYTH1	BASP1	FPR1	PLVAP	COL1A2
HMGB2	TNFRSF13B	CD79A	IL2RA	HAVCR2	CD84	SEMA4A	CD5	CD300LB	FASLG	CD300LF	FPR2	ECSCR	COL6A1
SSBP2	ZBTB32	BMS1P20	CD3E	CD244	CDK5R1	CD244	ENTPD1	CNR1	GRB2	DAZAP2	GPR77	SLCO2A1	COL6A2
PTTG1		ADAM6	CD47	CD160	FGF2	FASLG	CTLA4	CNR2	KLRG1	EIF4A1	MAEA	CCL14	COL6A3
BACH2		NUGGC	EOMES		GPR18	TNFSF4	IZUMO1R	CPM	LILRB5	EIF4G2	PROK2	MMRN1	CXCL14
STAG3		MEI1	GNLY		PDCD1	C1orf21	TNFRSF18	CSF3R	LST1	EMP3	SEC14L1	MYCT1	LUM
PPAPDC1B		CD79B	GZMA			RGS13	IL2RA	ENG	MAPK4	FCN1	SEPX1	KDR	COL3A1
CD72		PAX5	GZMB			NR5A2	ITGAE	FCAR	NOTCH3	FTL	SLC25A37	TM4SF18	DPT
LRMP		FCER2	PRF1			SNAP47	LAG3	IGF1	PIK3CG	GABARAP	TNFSF14	TIE1	ISLR
SYNE2		FCRL2	IFNG			GALNT2	TGFB1	IL34	PILRA	HIF1A	TREML4	ERG	PODN
IGHD		CXCR5	CD8A			LYST	LRRC32	L1CAM	PLCG2	LITAF	VNN2	FABP4	CD248
GCHFR		CD19	CD8B			AC092580.4	TNFRSF4	LILRA1	SIGLEC7	NCOA4	XPO6	SDPR	FGF7
ELL3		FCRLA	CD95L			FAM49A	SELL	LILRA5	SIGLEC9	OLR1		HYAL2	MXRA8
NEIL1		BLK	LAMP1			FAM179A	FOXP3	LRP1		RAB1A		FLT4	PDGFRL
MCTP2		FCRL1	LAG3			CRIM1	STAT5A	MS4A7		RHOA		EGFL7	COL14A1
RP11-231C14.7		MS4A1	CTLA4			GNLY	STAT5B	MS4A8B		SAT1		ESAM	MFAP5
DEF8		CNR2	HLA-DRA			CD8A	LGALS1	TREM1		SDCBP		CXorf36	MEG3
PRPSAP2		BANK1	TNFRSF4			CD8B	IL10	TREML1		SRGN		TEK	SULF1
FCER2		TNFRSF13B	ICOS			GPAT2	IL12A			TEK		TSPAN18	AOX1
			TNFRSF9			NR4A2	EBI3			TMBIM6		EMCN	SVEP1
			TNFRSF18			GPD2	TGFB1			UBE2D3		MMRN2	LPAR1
						CERS6						ELTD1	PDGFRB
						TTN						PDE2A	TAGLN
						CCDC141						NOS3	IGFBP6
						ITM2C						ROBO4	FBLN1
						UBE2F						APOLD1	CA12
						PDCD1						PTPRB	SPOCK1
						SRGAP3						RHOJ	TPM2
						RP11-222K16.2						RAMP2	THBS2
						EOMES						GPR116	FBLN5
						CMC1						F2RL3	TMEM119
						CCR1						JUP	ADAM33
						CCR5						CCBP2	PRRX1
						ABHD6						GPR146	PCOLCE
						DZIP3						RGS16	IGF2
						CD200R1						TSPAN7	GFPT2
						GTPBP8						RAMP3	PDGFRA
						HEG1						PLA2G4C	CRISPLD2
						GOLIM4						TGM2	CPE
						GNB4						LDB2	F3
						CD38						PRCP	MFAP4
						DTHD1						ID1	C1S
						STAP1						SMAD1	PTGIS
						TNIP3						AFAP1L1	LOX
						TMEM155						ELK3	CYP1B1
						ITGA1						ANGPT2	CLDN11
						ITGA2						LYVE1	SERPINF1
						GZMA						ARHGAP29	OLFML3
						PLPP1						IL3RA	COL5A2
						RASA1						ADCY4	ACTA2
						PDLIM4						TFPI	MSC
						TIMD4						TNFAIP1	VASN
						HAVCR2						SYT15	ABI3BP
						DBN1						DYSF	C1R
						GFOD1						PODXL	ANTXR1
						AIF1						SEMA3A	MGST1
						HLA-DRA						DOCK9	C3
						HLA-DRB5						F8	PALLD
						HLA-DRB1						NPDC1	FBN1
						HLA-DQA1						TSPAN15	CPXM1
						HLA-DQB1						CD34	CYBRD1
						SOBP						THBD	IGFBP5
						VNN2						ITGB4	PRELP
						CHST12						RASA4	PAPSS2
						ETV1						COL4A1	MMP2
						CHN2						ECE1	CKAP4
						WIPF3						GFOD2	CCDC80
						TRGV9						EFNA1	ADAMTS2
						TRG-AS1						PVRL2	TPM1
						LIMK1						GNG11	PCSK5
						PON3						HERC2P2	ELN
						PON2						MALL	CXCL12
						LRRN3						HERC2P9	OLFML2B
						FAM3C						PPM1F	PLAC9
						ADAM28						PKP4	RCN3
						TOX						LIMS3	LTBP2
						ASPH						CD9	NID2
						MSC						RAI14	SCARA3
						FABP5						ZNF521	AMOTL2
						MTSS1						RGL2	TPST1
						PIP5K1B						HSPG2	MIR100HG
						RP11-305L7.1						TGFBR2	CTGF
						RP11-305L7.3						RBP1	RARRES2
						SLC2A8						FXYD6	FHL2
						RP11-492E3.2						MATN2	
						SLC2A6						S1PR1	
						DBH-AS1						PIEZO1	
						CLIC3						PDGFA	
						PNPLA7						ADAM15	
						PRR5L						HAPLN3	
						PLA2G16						APP	
						CTSW							
						TPCN2							
						MYO7A							

PRSS23
DIXDC1
FXYD2
CRTAM
BARX2
NEBL
SPAG6
PRF1
ENTPD1
PIK3AP1
AFAP1L2
CD9
LPAR5
PTMS
LAG3
CLECL1
CLEC2B
KLRD1
RP11-
KLRK1
KLRC4
KLRC2
KLRC1
RP11-291B21.2
NELL2
KRT86
KRT81
ITGB7
CD63
IFNG
TMTC2
HVCN1
KATNAL1
SERP2
TSC22D1
SPRY2
KDELC1
GZMH
GZMB
AKAP5
FUT8
DPF3
ATP8B4
DAPK2
PIF1
SEMA7A
MIR9-3HG
MCTP2
LRRC28
GTF3C1
NOD2
CPNE2
ADGRG5
ADGRG1
GSG2
RASD1
AC069363.1
CCL3
CCL4
CCL3L3
CCL4L2
LINC00672
RP11-
GNGT2
ABI3
SCPEP1
PECAM1
FBF1
GALNT1
BCAS4
ZFP82
LINC01480
ATP1A3
NKG7
KIR2DL4
DGCR6
KIAA1671
HMOX1
APOBEC3H
LINC00158
MIR155HG

Supplementary Table 9. Cell numbers of the 15 T cell clusters according to the sample of origin

	TNBC2	TNBC3	TNBC4	TNBC5	TNBC6	PBMC2	PBMC3	PBMC4	PBMC5	PBMC6
C1	71	18	8	18	1369	110	17	38	6	16
C2	619	183	9	14	622	75	8	33	20	32
C3	767	41	3	0	222	107	84	34	38	39
C4	11	9	1	2	77	521	40	439	128	46
C5	28	2	2	1	44	229	448	148	170	120
C6	19	15	0	5	73	410	33	180	115	67
C7	89	44	7	4	619	28	14	9	11	10
C8	198	24	3	14	423	11	60	10	30	11
C9	36	11	0	7	428	32	128	39	24	50
C10	66	14	1	31	406	13	42	12	25	13
C11	7	3	1	4	19	175	81	69	183	52
C12	296	43	3	6	199	11	9	15	5	2
C13	117	112	1	11	157	11	2	2	3	1
C14	3	2	0	19	53	91	25	22	109	24
C15	34	9	1	6	90	53	17	8	13	6

Supplementary References

1 Oh, D. Y. *et al.* Intratumoral CD4+ T cells mediate anti-tumor cytotoxicity in human bladder cancer. *Cell* **181**, 1612-1625. e1613 (2020).

2 Miao, Y. R. *et al.* ImmuCellAI: a unique method for comprehensive T-cell subsets abundance prediction and its application in cancer immunotherapy. *Advanced science* **7**, 1902880 (2020).

3 Duhen, T. *et al.* Co-expression of CD39 and CD103 identifies tumor-reactive CD8 T cells in human solid tumors. *Nature communications* **9**, 1-13 (2018).

4 Savas, P. *et al.* Single-cell profiling of breast cancer T cells reveals a tissue-resident memory subset associated with improved prognosis. *Nature medicine* **24**, 986-993, doi:10.1038/s41591-018-0078-7 (2018).

5 Butler, A., Hoffman, P., Smibert, P., Papalexi, E. & Satija, R. Integrating single-cell transcriptomic data across different conditions, technologies, and species. *Nature biotechnology* **36**, 411-420, doi:10.1038/nbt.4096 (2018).

6. Briney, B., Inderbitzin, A., Joyce, C. & Burton, D. R. Commonality despite exceptional diversity in the baseline human antibody repertoire. *Nature* **566**, 393-397, doi:10.1038/s41586-019-0879-y (2019).

7 Iglesia, M. D. et al. Prognostic B-cell signatures using mRNA-seq in patients with subtype-specific breast and ovarian cancer. Clinical cancer research **20**, 3818-3829 (2014).

8 Azizi, E. et al. Single-Cell Map of Diverse Immune Phenotypes in the Breast Tumor Microenvironment. Cell **174**, 1293-1308 e1236, doi:10.1016/j.cell.2018.05.060 (2018).

9 Angelova, M. et al. Characterization of the immunophenotypes and antigenomes of colorectal cancers reveals distinct tumor escape mechanisms and novel targets for immunotherapy. Genome biology **16**, 1-17 (2015).

10 Tirosh, I. et al. Dissecting the multicellular ecosystem of metastatic melanoma by single-cell RNA-seq. Science **352**, 189-196, doi:10.1126/science.aad0501 (2016).
