# Supplementary material for: Atlas of breast cancer infiltrated B-lymphocytes revealed by paired single-cell RNA-sequencing and antigen receptor profiling
Source: Nat Commun. 2021 Apr 12;12:2186. doi: 10.1038/s41467-021-22300-2 (PMC8042001; doi:10.1038/s41467-021-22300-2)
Supplement: Supplementary file 3 — Description of Additional Supplementary Files [file 41467_2021_22300_MOESM3_ESM.pdf]

Description of Additional Supplementary Files

File Name: Supplementary Data 1
Description: Data sheet 'single productive *IGH* B cells' were 11,732 B cells with single productive *IGH*. Data sheet 'transcriptome and single *IGH*' were 8,632 B cells with both single productive *IGH* and transcriptome data, together with their tSNE coordinates and their subgroup information.

File Name: Supplementary Data 2
Description: Marker genes of the 4 B cell clusters. These genes were identified by comparing their expression in a cluster with all other B cell clusters using 'FindAllMarkers' function in Seurat R package. The statistical test used was two-sided wilcoxon rank sum test, and p-value adjustment was performed using bonferroni correction

File Name: Supplementary Data 3
Description: Marker genes of the 13 B cell clusters. These genes were identified by comparing their expression in a cluster with all other B cell clusters using 'FindAllMarkers' function in Seurat R package. The statistical test used was two-sided wilcoxon rank sum test, and p-value adjustment was performed using bonferroni correction

File Name: Supplementary Data 4
Description: Survival analysis in METABRIC dataset for breast cancer subtypes. The statistical test used was two-sided log-rank test without correction for multiple testing.

File Name: Supplementary Data 5
Description: Survival analysis in TCGA samples. The statistical test used was two-sided log-rank test without correction for multiple testing.

File Name: Supplementary Data 6
Description: Marker genes for 15 T cell clusters. These genes were identified by comparing their expression in a cluster with all other T cell clusters using 'FindAllMarkers' function in Seurat R package. The statistical test used was two-sided wilcoxon rank sum test, and p-value adjustment was performed using bonferroni correction

File Name: Supplementary Data 7
Description: Gene Set Enrichment Analysis (GSEA) on differentially expressed genes between TNBC B cells and PBMC B cells. The statistical test used was one-sided Kolmogorov-Smirnov test and adjustments were made for multiple comparisons in the FDR column.
